# Built different: ER cisternae formed by the Arabidopsis Lunapark proteins differ in ultrastructure and affect ER–Golgi transport

**DOI:** 10.1111/nph.71217

**Published:** 2026-04-29

**Authors:** Charlotte Pain, Tatiana Spatola Rossi, Nadine Field, Carmen Mata, Alessia Candeo, Muhammad W. Ali, Flavia Moreira‐Leite, Stanley W. Botchway, Federica Brandizzi, Verena Kriechbaumer

**Affiliations:** ^1^ Endomembrane Structure and Function Research Group, School of Biological and Medical Sciences, Oxford Brookes University Gipsy Lane Oxford OX3 0BP UK; ^2^ Oxford Brookes Centre for Bioimaging, Oxford Brookes University Gipsy Lane Oxford OX3 0BP UK; ^3^ Dipartimento di Fisica Politecnico di Milano Piazza Leonardo da Vinci 32 20133 Milan Italy; ^4^ Central Laser Facility, Science and Technology Facilities Council, Research Complex at Harwell, Rutherford Appleton Laboratory Didcot OX11 0QX UK; ^5^ MSU‐DOE Plant Research Lab, Michigan State University East Lansing MI 48824 USA; ^6^ Department of Plant Biology Michigan State University East Lansing MI 48824 USA; ^7^ Great Lakes Bioenergy Research Center, Michigan State University East Lansing MI 48824 USA

**Keywords:** AnalyzER, *Arabidopsis thaliana*, electron tomography, endoplasmic reticulum, ER structure, ER–Golgi transport, Lunapark, *Nicotiana tabacum*, transmission electron microscopy

## Abstract

- The plant endoplasmic reticulum (ER) is a dynamic organelle composed of multiple distinct structural domains, such as cisternae, which are maintained by ER morphogens including the *Arabidopsis thaliana* Lunapark proteins (LNPs). Cisternae are typically described as sac‐like structures connected by tubules. Here we challenge this assumption and propose that cisternae have a more complex structure that modifies ER functionality.
- This study used state‐of‐the‐art high‐resolution confocal and variable‐angle epifluorescence microscopy, along with transmission electron microscopy and tomography, on high‐pressure frozen *Arabidopsis thaliana* samples.
- We found that AtLNP1‐stabilised ER forms cisternae composed of dense tubular matrices, whereas AtLNP2 forms cisternae with a uniform, sac‐like structure. Furthermore, overexpression of AtLNP proteins alters Golgi morphology, affecting ER‐to‐Golgi transport and secretion.
- Our findings reveal that the balance between AtLNP1 and AtLNP2 is critical for ER cisternae organisation and ER functionality in protein production and secretion. This work provides new insights into ER structural plasticity and its functional implications in plant cells.

## Graphical abstract

The endoplasmic reticulum (ER) is a key structure in plant cells that helps produce and transport proteins. We discovered that different Lunapark proteins shape ER structures in distinct ways, affecting protein movement and secretion. Understanding how ER structure influences function can help improve plant‐based production of valuable compounds, such as medicines or bio‐based materials.

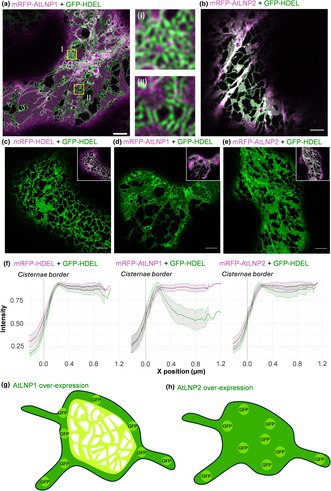

## Introduction

In plants, the endoplasmic reticulum (ER) forms a network with two distinct structural domains: tubules and cisternae. Tubules are narrow structures (*c*. 40 nm in diameter; Pain *et al*., 2019) that join at three‐way junctions, whereas cisternae are described as expanded, flattened, sac‐like regions connected to tubules (Westrate *et al*., 2015; Griffing *et al*., 2017). Recent studies in mammalian cells challenge this simplified view, suggesting that cisternae may exhibit a range of structures ranging from smooth sac‐like cisternae near the nucleus to dense tubular matrices at the cell periphery (Nixon‐Abell *et al*., 2016). However, this aspect of cisternal substructure has not been explored in plants.

The ER is involved in key cellular functions, including protein synthesis, folding and quality control (Jan *et al*., 2014), lipid synthesis (Jacquemyn *et al*., 2017) as well as metabolite storage (Chen *et al*., 2002; Zhang *et al*., 2013) and metabolon formation (Hawes *et al*., 2015). Despite the importance of these processes, little is known about the functional roles of the different ER structures. Studies have shown a correlation between increased cisternal area and enhanced secretory capacity in plant and mammalian cells (Stephenson & Hawes, 1986; Ridge *et al*., 1999; Stefano *et al*., 2015). In plants, the precise proportions of tubules and cisternae are in constant flux, driven by rapid ER remodelling (Sparkes *et al*., 2009a, 2009b; Pain *et al*., 2019; Pain & Kriechbaumer, 2020). However, the overall proportion of cisternae and tubules is consistent and linked with cellular development and biosynthetic requirements (Stephenson & Hawes, 1986).

Three main protein families regulate the structure of the plant ER: the reticulon family (RTN), Root Hair Defective 3 (RHD3) and the Lunapark (LNP) proteins (Tolley *et al*., 2008; Sparkes *et al*., 2010; Breeze *et al*., 2016; Kriechbaumer *et al*., 2018; Ueda *et al*., 2018). Reticulon proteins induce positive membrane curvature, essential for maintaining tubular structures (Tolley *et al*., 2010), while RHD3 acts as a putative ER fusogen (Chen *et al*., 2011; Sun & Zheng, 2018). In mammals, the LNP protein stabilises three‐way junctions, although it does not induce cisternae formation (Chen *et al*., 2015). In Arabidopsis, two LNP proteins, AtLNP1 and AtLNP2, localise differently within the ER, with AtLNP1 specifically targeting cisternae. Increased AtLNP1 and AtLNP2 expression results in an increase in cisternal area, whereas their loss reduces it (Kriechbaumer *et al*., 2018; Sun *et al*., 2020). AtLNP proteins also interact with RHD3 at three‐way junctions, potentially inhibiting RHD3's fusogenic function by promoting its degradation (Sun *et al*., 2020).

Not all mammalian ER morphogens have plant homologues. For example, Climp‐63, a mammalian protein that induces cisternae proliferation, does not have a counterpart in plants (Sandoz & van der Goot, 2015). Additionally, plant‐unique proteins like SYP73 have been suggested to act as connectors between the ER membrane and actin (Cao *et al*., 2016), indicating that plants may use specific strategies to maintain ER structure.

Understanding the mechanisms behind the formation of distinct ER structural domains in plants is challenging, requiring advanced imaging techniques. Using dynamic, high‐resolution confocal microscopy and electron microscopy, we found that AtLNP1 and AtLNP2 produce distinct ER cisternae: AtLNP1 forms cisternae with dense tubular matrices, whereas AtLNP2 creates sac‐like structures. These findings are supported by both transient and stable overexpression (OE) in tobacco and Arabidopsis leaves and roots. These findings challenge previous assumptions about cisternal morphology and reveal that AtLNP proteins affect ER functionality, including biosynthetic capacity. Overexpression of AtLNP proteins impairs ER‐to‐Golgi transport and secretion, with altered Golgi body morphologies, highlighting the importance of balanced cisternal structures for efficient secretory function. Interestingly, protein production capacity was impaired only upon AtLNP2 OE, suggesting that different AtLNP proteins have specific roles in regulating ER function.

## Materials and Methods

### Constructs and stable lines

Constructs and stable *Arabidopsis thaliana* (L.) Heynh. lines used in this work were originally generated in previously published studies (Supporting Information Table S1). roGFP2‐AtLNP1, roGFP2‐AtLNP2, AtLNP1‐roGFP2 and AtLNP2‐roGFP2 were produced via Gateway cloning using the PCMO1 and pSS01 destination vectors for N‐terminal and C‐terminal fusions, respectively (Brach *et al*., 2009). A plasmid containing antiF4 was obtained using TWIST Bioscience (Twist pENTR) and then cloned into a plant expression vector (pB7FWG2, Karimi *et al*., 2002) using Gateway cloning technology.

### Transient expression and plant materials

Transient protein expression was performed by Agrobacterium‐mediated transformation of *Nicotiana tabacum* L. (SR1 cv Petit Havana). *Nicotiana tabacum* plants were grown in a glasshouse at 23°C with a 12‐h light : dark cycle. Transformation of *N. tabacum* was performed as described previously, using the *Agrobacterium tumefaciens* strain GV3101 (Sparkes *et al*., 2006). Briefly, transformed agrobacteria were pelleted by centrifugation at 2200 ***g*** at room temperature for 5 min. The pellet was then washed once with infiltration buffer (5 mg ml^−1^ glucose, 50 mM MES, 2 mM Na_3_PO_4_·12H_2_O and 0.1 mM acetosyringone in deionized water) and then resuspended. The bacterial suspension was then diluted to the required optical density (in this case OD_600_ = 0.1) and then injected into the abaxial side of the tobacco leaf using a syringe with no needle. Infiltrated plants were then placed in an incubator for 3 d at 23°C with a 12‐h light : dark cycle before imaging.

### Confocal microscopy

Approximately 5 mm^2^ pieces of infiltrated tobacco leaves were cut with a sharp razor blade and immediately mounted in water before imaging. Tobacco leaf epidermal cells were imaged using either a Zeiss PlanApo ×100/1.46 NA oil immersion objective or PlanApo 63×/1.46 NA oil immersion objective on a Zeiss 880 LSM confocal with Airyscan detector. eGFP was excited using a 488 nm laser and emission was detected at 523 nm. mRFP was excited using a 561 nm laser and emission was detected at 579 nm. Laser power is detailed in Table S2. All images were collected in line switch scanning mode with 2–4× averaging. Pixel scaling was typically 0.046 μm^2^ and frame rate of 10.7 s.

### RNA extraction

For tobacco RNA‐seq, leaf samples from 3 independent plants and infiltration events were prepared 3 d after infiltration using a scalpel and ground to a powder using prechilled mortar and pestles with liquid nitrogen. For Arabidopsis RNA‐seq, three independent seedling batches were grown for 10 d. Samples were ground to a powder using prechilled mortar and pestles with liquid nitrogen. Total RNAs from samples frozen in liquid nitrogen were extracted using the Maxwell RSC Plant RNA Kit (FB193; Promega, Madison, WI, USA) following the manufacturer's protocol. RNAs were solubilized in DNAse‐free water and quantified using a Qubit RNA High Sensitivity Broad Range Assay Kit (Thermo Fisher Scientific, Waltham, MA, USA). RNA quality was assessed using RNA ScreenTape Assay (Agilent, Santa Clara, CA, USA). RNA was stored at −80°C until sequencing. From each sample, 10 μg of RNA were sent to Novogene (Cambridge, UK) for sequencing to a depth of 20 million reads using the Illumina Novaseq X Plus PE150 platform. The sequencing quality of the files from the RNA‐seq data was examined by FastQC software (v.0.10.1; http://www.bioinformatics.babraham.ac.uk/projects/fastqc/). RNA‐seq reads were created and processed to analyse gene transcript levels.

### Mapping reads and differentially expressed genes (DEGs) analysis

All analyses were performed in R Studio (v.1.4) with R (v.4.4.1) (R Core Team, 2013). All R codes for the DEG analyses are available upon request. The clean reads of sequenced data were mapped to the *N. tabacum* reference genome with default parameters using HISAT2 (v.2.2.0) (Kim *et al*., 2019). Only uniquely mapped reads to the transcripts were calculated with the featureCounts algorithm (v.2.0.2) (Liao *et al*., 2014). DESeq2 R package (Anders & Huber, 2010; Love *et al*., 2014) were used to identify DEGs with a filter log_2_FC > 1 and with an adjusted *P*‐value of < 0.05. Transcript abundance was estimated using transcript‐level quantification software capable of generating transcripts per million (TPM) values. TPM matrices were extracted for all endogenous tobacco LNP‐like genes and the AtLNP1/AtLNP2 transgenes. Log_2_‐transformed TPM values were used for visualization.

### Cisternae structure analysis

Identification and analysis of ER cisternae was performed as described by Pain *et al*. (2019), full details are available in the accompanying manual (DOI: 10.5281/zenodo.6386982). In brief, images produced as described above (see ‘Confocal microscopy’ in the Materials and Methods section) were imported into AnalyzER software (Pain *et al*., 2019), and the minimum and maximum full width at half maximum ‘FWHM’ measurements, which determine the minimum and maximum widths of ER tubules, were then set. After upsampling of the image to prevent later pixelation artefacts, background subtraction and filters to improve contrast were applied. The ER was segmented using hysteresis thresholding, and cisternae were identified using an opening function.

Once cisternae were identified, further analysis could be performed either on both image channels or on a single channel. The distribution of GFP‐HDEL through cisternae was analysed across both channels by separating identified cisternae into radial bins. The intensity along each radial section was measured and then averaged for each cisterna to give a radial intensity profile. These radial intensity measurements were then averaged across all cisternae in all cells to give a mean radial plot. A minimum of five cisternae per image is required before averaging to ensure a representative sample has been collected.

Grey‐level co‐occurrence matrix (GLCM) analysis was performed only on the GFP‐HDEL (lumenal ER marker) channel to measure disruptions in lumenal contents distribution. The GLCM of each cisterna was generated by creating an accumulator array comparing each cisternae pixel to neighbouring pixels, the radius of which is user‐defined. We set the neighbourhood size of the GLCM at the approximate width of a single tubule. The radius of tubules is calculated as in Pain *et al*. (2019); briefly, the radius of a tubule is assumed to be the same approximate width as a cisterna, calculated by incorporating the point spread function (psf) of the microscope (thought to be *c*. 140 nm) and the ratio of intensities between the tubules and cisternae.

The various properties of the GLCM were calculated using the graycoprops command in MATLAB. Four GLCM properties are commonly analysed: contrast, correlation, energy and homogeneity. These properties were measured for each cisterna and then summarised for all the cisternae pixels in the network to prevent results from being dominated by many smaller cisternae. Of the three properties that were of particular interest, cisternal contrast measures the contrast between each pixel and its neighbours over an image. A value closer to 0 represents a more constant image. Cisternal energy is a sum of the squared GLCM elements, and a value of 1 is expected for a constant image. Cisternal homogeneity measures the distribution of elements in the GLCM diagonal. A diagonal GLCM will have a value of 1. Full details of the GLCM property analysis are included in Table S3.

It should be noted this technique is designed to work with lower resolution images, with an estimated lateral resolution of *c*. 120 nm (Pain *et al*., 2019), compared to other published techniques that have been described at *c*. 100 nm resolution (Nixon‐Abell *et al*., 2016) and 50 nm resolution, respectively (Schroeder *et al*., 2019).

For statistical analysis, an initial MANOVA was carried out. Subsequent comparisons were performed using ANOVAs for each texture metric with a Bonferroni correction applied to identify groups with significant differences. Finally, Tukey HSD was applied to identify differences from control. *n* numbers are detailed in figure legend.

### Topology analysis using redox‐sensitive GFP (roGFP2)

roGFP2 constructs were infiltrated into *N. tabacum* plants and imaged at 2 d after infiltration. Images were acquired on a PlanApo 63×/1.46 NA oil immersion objective, with excitation at 405 nm and 488 nm and detection at 500–555 nm, and excitation at 405 nm with detection at 410–470 nm for autofluorescence measurements, using three tracks with line switching. Full details of the laser power used are included in Table S2. The pixel‐by‐pixel ratio for each image was obtained using the batch processing mode of a specialized redox ratio analysis software (Fricker, 2016), following instructions in the manual. In total, three biological replicates (plants) and 10 technical replicates (cells) per plant were imaged per construct. The mean and SD are shown for the 30 measurements.

### Variable angle epifluorescence microscopy (VAEM)

VAEM was performed using a Zeiss Axio‐Observer z1 with a 100×/1.46 NA oil immersion objective. The attached optical system was the iLas2 TIRF system which provides orbital excitation, resulting in even illumination across the field of view. Laser power was set to *c*. 5 mW (continuous wave) and an excitation wavelength of 488 nm was used. Images were collected using an Andor iXon cooled EMCCD camera. Effective pixel size was 160 nm with an exposure time of 50 ms.

### High pressure freezing and electron microscopy

Arabidopsis seeds were sterilised using 70% ethanol for 3 min, dried and then transferred to ½‐strength Murashige & Skoog plates which were placed in a fridge at 4°C in the dark for 3 d. They were then transferred to a plant incubator set to 21°C on a long‐day light cycle for 10 d. To prepare samples for high pressure freezing (HPF), root tips were cut using microdissection scissors and then transferred to aluminium planchettes containing 20% BSA in PBS. Fixation of root tissue for the TEM micrographs and electron tomography of Golgi bodies was performed by high pressure freezing (HPF) in a Bal‐Tec HPM 010 (Abra Fluid AG). Fixation of tissue for the electron tomography of cisternae was performed using HPF in an EM ICE (Leica Microsystems, Wetzlar, Germany). Samples were then transferred to an EM AFS2 freeze substitution system (Leica Microsystems) in cryovials with a freeze substitution cocktail of 2% osmium and 0.1% uranyl acetate in acetone. Samples were subjected to a 90‐h freeze substitution programme, consisting of the following steps: 12 h at −90°C; −90°C to −85°C over 6 h; −85°C to −20°C over 48 h; 12 h at −20°C; −20°C to 10°C over 12 h. Samples were then transferred to room temperature, washed in 100% acetone and then further stained with 2% osmium in acetone at room temperature for 1 h. After the second osmium step, samples were washed in 100% acetone and then infiltrated and embedded in hard epoxy resin (TAAB 812 hard), with a total infiltration period of 4 d. Ultra‐thin sections of 70 nm were collected using a PowerTome ultramicrotome (RMC), poststrained with Reynolds' lead citrate and then imaged at 120 kV in a JEOL JEM‐1400Flash (JEOL) transmission electron microscope equipped with Gatan OneView 16‐megapixel camera (Gatan – Ametek). Electron tomography was performed on a 150 nm section collected onto formvar‐coated slot grids. Grids were mounted in a Fischione dual‐axis tomography holder (Fischione instruments), and dual‐axis tilt‐series (55 to −55°; 1° tilt between images) were acquired using SerialEM (Mastronarde, 2005). Tomogram generation was performed in ETomo (IMOD software package; https://bio3d.colorado.edu/imod/), and the tomogram was segmented manually in 3dmod (also from the IMOD package), to produce the 3D model.

### Quantifying changes to Golgi body structure

Golgi body curvature on EM images was measured using the Kappa plugin (Mary & Brouhard, 2019) in Fiji (Schindelin *et al*., 2012). Curve fitting was done partially manually by selecting points along the edge of a Golgi body cisternae. These points were then joined to create a B‐spline curve, and the average curvature was measured for each stack (Fig. S1a). Golgi body stack widths were measured using the Integrated Distance Fiji macro. This macro measures the distance between two polylines using the shortest distances in pixels from a point along the shorter line to the longer line (Fig. S1b). Statistical analysis was carried out as follows. Initial normality testing showed a nonparametric test was more appropriate, therefore, a Kruskal–Wallis test was used. A Dunn test was then applied to find the difference between groups.

### Transport between the ER and the Golgi bodies

For each cell analysed, Golgi body intensity was measured by drawing an region of interest (ROI) on three Golgi bodies, ER intensity was measured by drawing three ROIs on the ER, and a background intensity measurement was taken from just outside the cell. The average Golgi body intensity and ER intensity were taken from these ROIs, and the background intensity subtracted from both. The percentage intensity of Golgi bodies was calculated in comparison to total intensity across the ER and Golgi bodies. These measurements were collected from each technical replicate (cells), of which RFP‐HDEL *n* = 26, CXN‐mCherry *n* = 25, RFP‐AtLNP1 *n* = 39, and RFP‐AtLNP2 *n* = 23. Results are taken from four biological replicates (plants). For statistical analysis, a Kruskal–Wallis test was applied as initial normality testing showed a nonparametric test was appropriate.

### Secretion of SP‐mCherry to the apoplast

Secretion to the apoplast was quantified by measuring the ratio of the secretory pathway marker SP‐mCherry (da Costa *et al*., 2010) at the apoplast vs the interior of the cell 3 d postinfiltration into *N. tabacum* leaf epidermal cells. Cells were imaged as described above (see ‘Confocal microscopy’ in the Materials and Methods section), and images were analysed in ImageJ by drawing a ROI around the edge of the cell, using the ER resident protein as a guide for the cell interior. The mean intensity of SP‐mCherry in this region, the cell interior, was then measured. To measure the intensity of SP‐mCherry in the apoplast, the ROI was then enlarged by 1.5 μm and the Brush tool was used to remove the centre of the ROI, corresponding to the cell interior, to give a measure of the mean intensity of SP‐mCherry fluorescence in the apoplast. For statistical analysis a Kruskal–Wallis test was applied as initial normality testing showed a non‐parametric test was appropriate.

### Measurement of fluorescence intensity for protein amount

Fluorescence intensity measurements were performed using ImageJ (https://imagej.net/). Confocal images were taken using conventional confocal microscopy with constant laser power, an average of 8 and 512 × 512 pixels. Images were first imported into ImageJ, where ROIs including the whole ER were selected. To measure the fluorescence intensity, the ‘Measure’ function in ImageJ was applied which provided quantitative data on the mean intensity values within the selected ROIs. For statistical analysis, *t*‐tests were conducted.

### Western blotting

Total protein was extracted from *N. tabacum* leaf tissue 3 d after Agrobacterium‐mediated infiltration by flash‐freezing excised leaf segments in liquid nitrogen and grinding to a fine powder. Powdered tissue was resuspended in extraction buffer (50 mM Tris–HCl pH 7.5, 150 mM NaCl, 1% Triton X‐100, 0.1% SDS, 1× protease inhibitor cocktail), incubated on ice for 30 min, followed by centrifugation at 13 000 ***g*** for 10 min at 4°C to remove insoluble debris. Protein concentration was determined by Nanodrop measurements, and equal amounts of total protein per sample were mixed with 4× Laemmli sample buffer supplemented with reducing agent and heated at 70°C for 10 min before electrophoresis. Equal volumes of protein extracts were resolved by SDS‐PAGE on 12% polyacrylamide gels and transferred onto PVDF membranes using a wet transfer system (BioRad). Membranes were blocked for 1 h at room temperature in 5% (w/v) nonfat milk dissolved in TBS‐T (20 mM Tris–HCl pH 7.6, 137 mM NaCl, 0.1% Tween‐20) and incubated overnight with primary antibodies diluted 1 : 1000 in TBS‐T. Anti‐GFP antibodies (ChromoTek, Planegg‐Martinsried, Germany) were used to detect antiF4‐eGFP. Following washing in TBS‐T, membranes were incubated with CY3 labelled secondary antibodies for 1 h at room temperature. The membranes were washed 3 × 5 min in TBS‐T. Fluorescent signals were detected using a fluorescence imaging system, and band intensities were quantified using ImageJ software. AntiF4‐eGFP signal intensities were normalised to control samples. Western blotting was performed on three independent biological replicates.

## Results

### When is a sheet not a sheet: AtLNP1 and AtLNP2 modify ER structure differently

There is still significant debate on the structure and formation of ER cisternae (Nixon‐Abell *et al*., 2016; Schroeder *et al*., 2018). To investigate how AtLNP1 and AtLNP2 affect ER architecture and cisternae formation/maintenance, we deployed both stable OE systems in Arabidopsis and transient OE in the widely used heterologous *N. tabacum* (hereafter referred to as tobacco) leaf epidermis expression system, a well‐established platform for high‐level, short‐term protein expression and subcellular localisation studies (Zhang & He, 2021; Naeem *et al*., 2023; Liu *et al*., 2025). Throughout this study, the term transient OE is used to distinguish this approach from endogenous or stable expression systems and reflects the substantially elevated transcript levels achieved following Agrobacterium‐mediated delivery of the AtLNP1 and AtLNP2 proteins in comparison to Arabidopsis transformed with At LNP1 or ATLNP2 in a stable manner (Fig. S2).

**Fig. 1:**
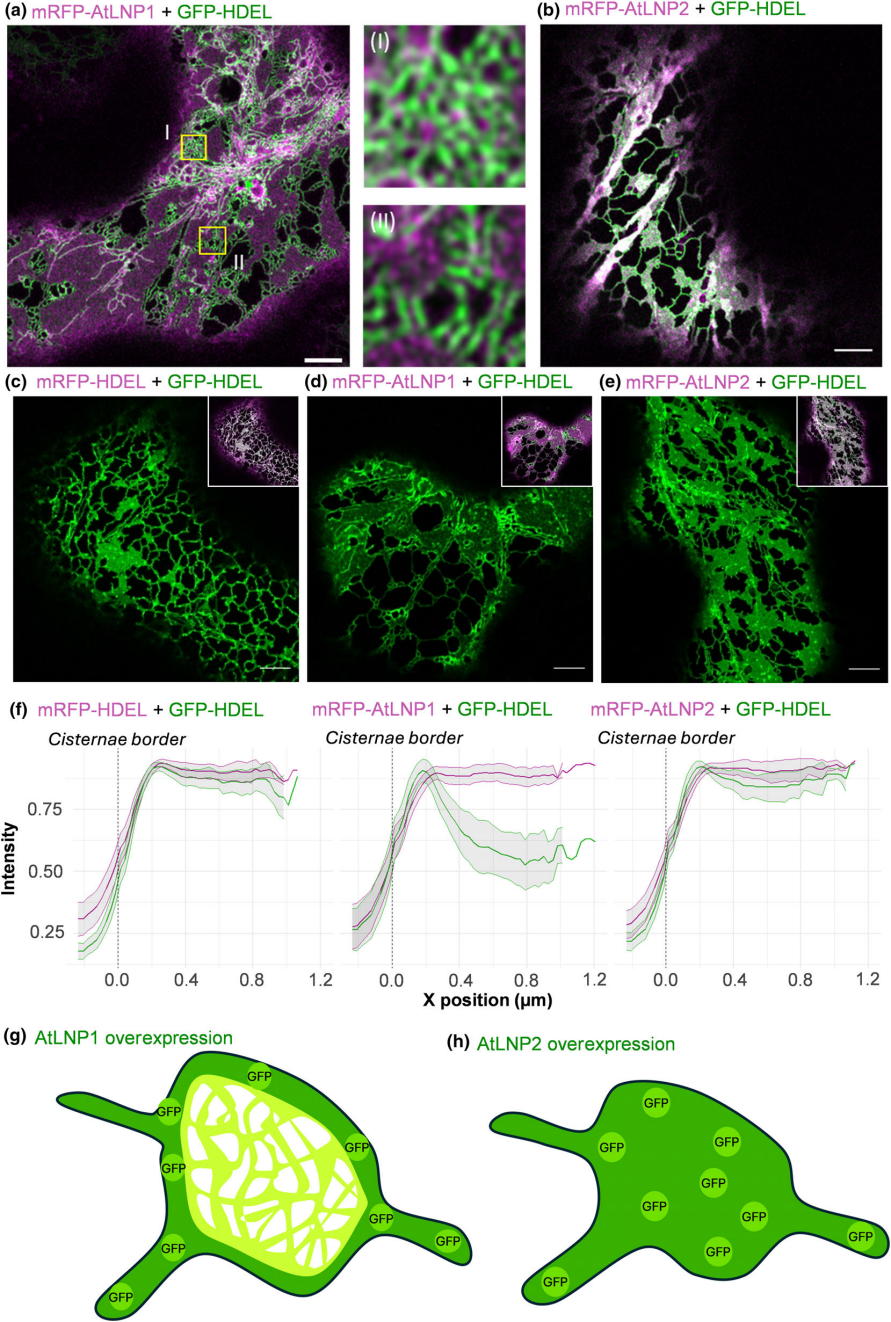
AtLNP1 and AtLNP2 form cisternae with distinct structures at the cisternal periphery and different lumenal characteristics. (a, b) Representative micrographs collected using confocal microscopy showing the unique morphology of endoplasmic reticulum (ER) cisternae induced by overexpression of mRFP‐AtLNP1/2 in *Nicotiana tabacum* leaf epidermal cells. Representative confocal images of overexpression (OE) of (a) mRFP‐AtLNP1 and (b) mRFP‐AtLNP2 (magenta) co‐expressed with the ER lumenal marker GFP‐HDEL (green). Images (I) and (II) are insets of areas highlighted by yellow boxes in a, showing details of two tubular matrices adjacent to mRFP‐AtLNP1‐induced cisternae. Bars, 5 μm. Single‐channel representative images of GFP‐HDEL (green) in cells overexpressing (c) mRFP‐HDEL, (d) mRFP‐AtLNP1, and (e) mRFP‐AtLNP2. Merged images are shown as an inset in the top right corner of each image. (f) Intensity plots of mRFP‐HDEL, mRFP‐AtLNP1 and mRFP‐AtLNP2 (magenta) alongside GFP‐HDEL (green), with SD plotted as a grey ribbon around the mean result. The vertical dashed line marks the edge of cisternae. Results are shown for three biological repeats with technical replicates of mRFP‐HDEL = 47, mRFP‐AtLNP1 = 54 and mRFP‐AtLNP2 = 40. Schematic representations of the distribution of the ER lumen‐targeted fluorophore GFP‐HDEL distribution across a cisterna induced by (g) AtLNP1 OE and (h) AtLNP2 OE. On AtLNP1 OE AtLNP1 may be excluded to the edge of the cisternae because of a dense tubular matrix making up the centre of the induced tubule. However, on AtLNP2 OE, GFP‐HDEL can access the entire cisternal lumen. Bars, 5 μm.

On transient OE, AtLNPs both induced the proliferation of large cisternae distributed throughout the ER which can be observed with the ER lumenal marker GFP‐HDEL (Kriechbaumer *et al*., 2018), composed of a fluorophore fused to a sporamin signal for ER targeting and the ER retention signal HDEL (Brandizzi *et al*., 2003). Furthermore, AtLNP1 OE had additional impacts on ER structure, namely the formation of many small, tightly clustered tubule bundles/nets formed around the edge of the induced cisternae (Fig. 1ai–ii). These net‐like structures were not formed on AtLNP2 OE (Fig. 1b), with the tubular network surrounding the cisternae having a more typical appearance. The evidence that ER structure surrounding cisternae is different on AtLNP1 and AtLNP2 OE raised the question of whether each forms cisternae with similar structural characteristics.

### LNP1 and LNP2 form cisternae with different lumenal characteristics

When the minimal ER lumenal marker GFP‐HDEL is expressed transiently and stably in most plant species, this marker will localise evenly throughout the interior of the ER (Fig. 1c,f). Indeed, measuring the distribution of GFP‐HDEL in radial transects across cisternae revealed an approximately even distribution of GFP‐HDEL across all cisternae. However, when co‐expressed with mRFP‐AtLNP1, GFP‐HDEL was localised primarily around the edge of cisternae and was largely excluded from the centre of cisternae (Fig. 1d,f). This was not the case on mRFP‐AtLNP2 OE, where GFP‐HDEL is distributed evenly throughout the lumen of the induced cisternae (Fig. 1e,f).

Taken together, these results indicate that AtLNP1 induces the formation of cisternae with an inaccessible lumen. These results, alongside the formation of dense tubular matrices abutting the edges of ER cisternae, lead us to infer that AtLNP1‐induced cisternae may be composed of dense tubular matrices with ER lumens that are tightly laterally compressed, limiting the flow of lumenal contents into the cisternae itself (Fig. 1g). This is not seen on the OE of AtLNP2 (Fig. 1h). Co‐expression of AtLNP1 and AtLNP2 together with the lumenal marker mTagBFP2‐HDEL resulted in a distribution of the HDEL marker throughout the cisterna (Fig. S3).

### AtLNP1 promotes negative membrane curvature

**Fig. 2:**
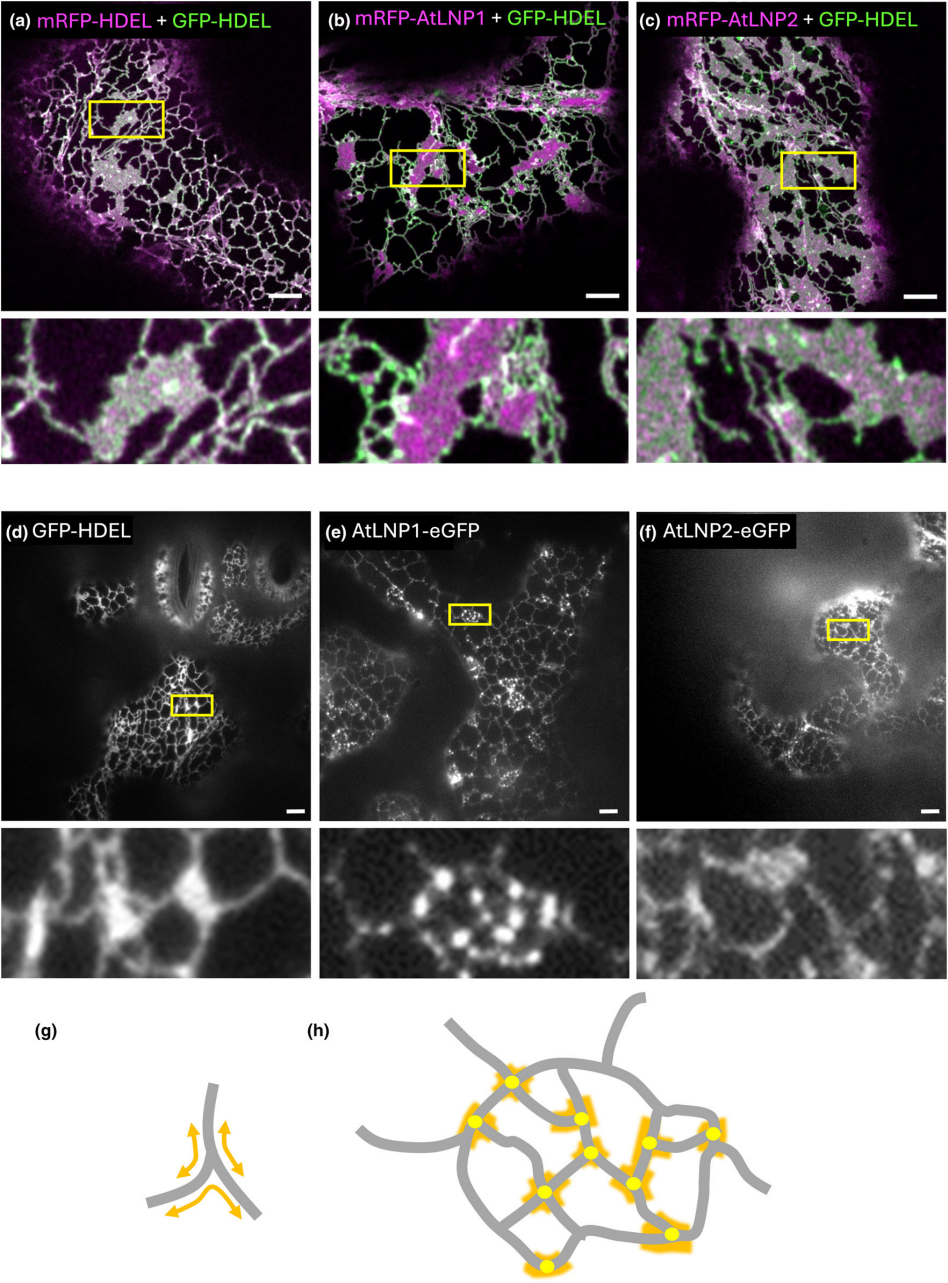
Dense tubular matrices are present on overexpression (OE) of AtLNP1 but are not present on AtLNP2 OE. (a–c) Representative image of transient OE in *Nicotiana tabacum* leaf epidermal cells of the endoplasmic reticulum (ER) lumenal marker mRFP‐HDEL alongside the (a) control GFP‐HDEL, (b) mRFP‐AtLNP1 and (c) mRFP‐AtLNP2 in tobacco leaf epidermal cells. Images collected using confocal microscopy, and yellow boxes show cisternal areas that are expanded below each image for clarity. (d–f) Representative stable OE in 10‐d‐old *Arabidopsis thaliana* cotyledonary cells, with images collected using variable angle epifluorescence microscopy. Images are shown for OE of (d) *P*_*35S*_::GFP‐HDEL, (e) *P*_*UBQ10*_::AtLNP1‐eGFP and (f) *P*_*UBQ10*_::AtLNP2‐eGFP. Insets of a similar size region to those selected for images a–c included alongside expanded images to show details of ‘cisternal’ areas. (g) Model of negative membrane curvature demonstrated around three‐way junction and (h) a model of a tubular net with the localisation of AtLNP1‐eGFP puncta shown in yellow, regions of negative membrane curvature highlighted in orange. Bars, 5 μm.

To investigate the structural changes in the ER controlled by AtLNP1 further, we investigated the effect of AtLNP1 on the ER when stably overexpressed in Arabidopsis. Stable expression of AtLNP1 and AtLNP2 (P_UBQ10_::AtLNP1‐eGFP and P_UBQ10_::AtLNP2‐eGFP, respectively) in Arabidopsis resulted in relatively low expression, requiring the use of particularly sensitive light microscopy techniques (variable angle epifluorescence microscopy, VAEM) to visualise the ER (Pain *et al*., 2024). Despite the relatively modest increase in transcript levels in the OE lines compared to endogenous AtLNP levels (Fig. S2), AtLNP1 OE still had a marked impact on the structure of the ER. These structures were different from those observed on transient expression in tobacco, where GFP‐HDEL expression was used as a control (Fig. 2a), mRFP‐AtLNP1 OE formed cisternae and dense tubular matrices (Fig. 2b), and mRFP‐AtLNP2 OE formed cisternae (Fig. 2c). GFP‐HDEL marked the entirety of the ER (Fig. 2d), as did AtLNP2‐eGFP (Fig. 2f). AtLNP1‐eGFP, however, in particular, localised to puncta at junctions present within dense tubular nets (Fig. 2e). In addition, cisternae could not be observed within the cells, making it likely that these dense tubular matrices had replaced these cisternae within the cell. These junctions are points of negative membrane curvature, where tubules bend at angles < 180° (Fig. 2g,h). AtLNP expression levels in Arabidopsis roots did not allow for VAEM imaging, so electron tomography of cryo‐fixed Arabidopsis roots was applied (Fig. S4). Also, here in the elongation zone of the roots, the ER in P_UBQ10_::AtLNP1‐eGFP transformants showed distinct puncta connected by tubular nets (Fig. S4a), whereas P_UBQ10_::AtLNP2‐eGFP transformants showed small unstructured cisternae (Fig. S4b).

Mammalian LNP induces the formation of densely branched tubules, with large aggregates of these tubules observed on increased expression to a point that individual tubules cannot be easily distinguished (Shemesh *et al*., 2014). This close packaging of ER tubules has been previously described in mammalian cells—whereby many cisternae are composed of dense tubular matrices. The tubules making up these cisternae rapidly assemble and disassemble, at such a rate that constant fusion and fission of tubules is not energetically favourable. Instead, the authors suggest that tubules coalesce until they become too small to observe using commercially available microscopy techniques due to both spatial and temporal limitations (Nixon‐Abell *et al*., 2016).

Therefore, we suggest that AtLNP1 may form cisternae of dense tubular matrices that are only visible using conventional microscopy systems around the edges of cisternae (Fig. 2b) or when the expression of AtLNP1 is significantly downregulated by the plant (Fig. 2e). These dense matrices often coalesce into a specific subtype of cisternae composed of tubular meshes.

### AtLNP2 OE also contributed to cisternae with an altered structure

**Fig. 3:**
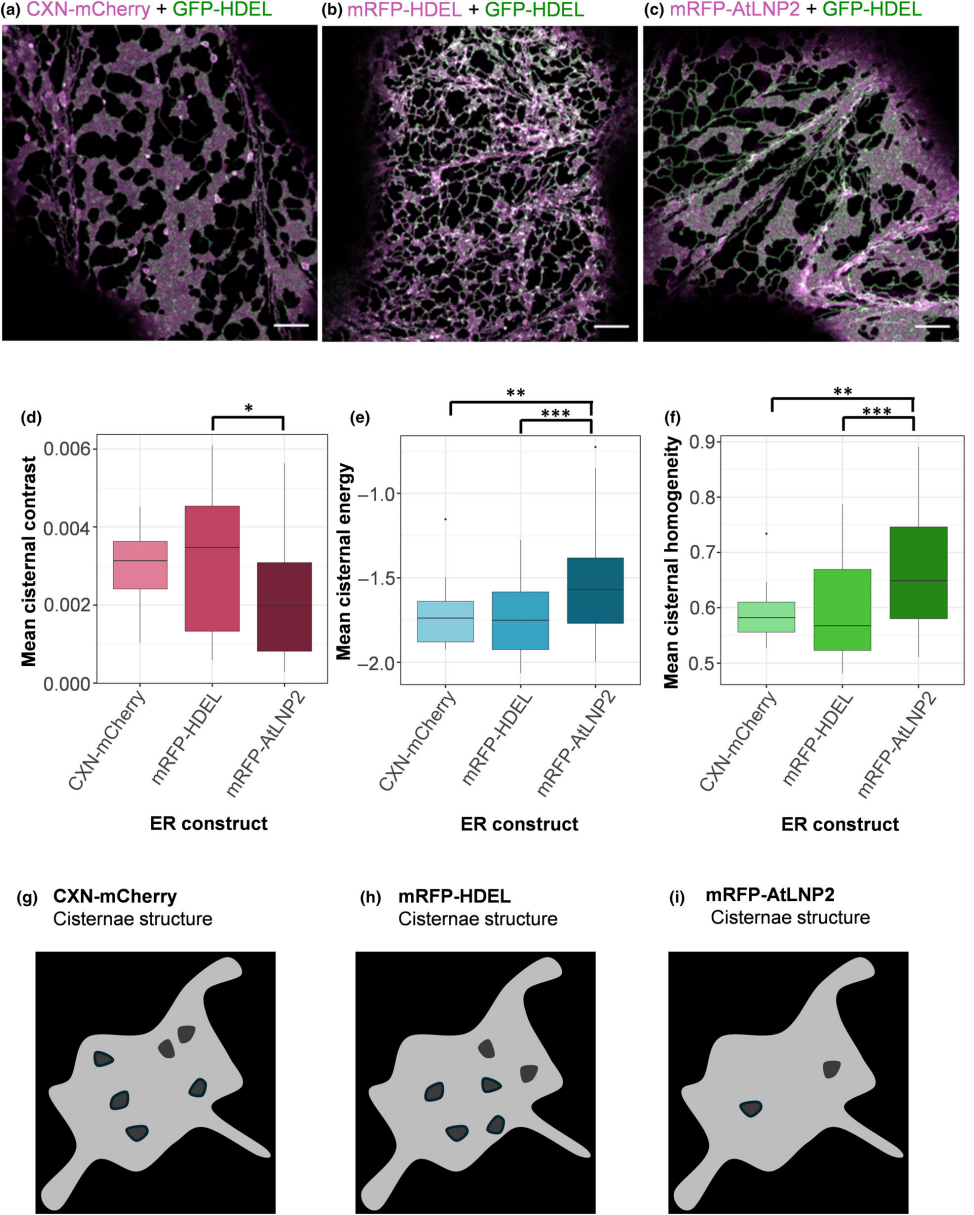
AtLNP2‐induced cisternae have unique lumenal characteristics that can be detected using image texture analysis. Representative images (*Nicotiana tabacum* leaf epidermal cells) of the endoplasmic reticulum (ER) membrane marker (a) CXN‐mCherry, (b) the ER lumenal marker mRFP‐HDEL and (c) the ER morphogen mRFP‐AtLNP2 (magenta) are shown co‐expressed with GFP‐HDEL (green). Boxplots comparing the mean cisternal contrast (d), energy (e) and homogeneity (f). Boxplots show median (centre line), interquartile range (box) and whiskers extending to 1.5× inter‐quartile range; outliers are plotted as individual points. Images collected using confocal microscopy with Airyscan. Results were taken from three biological repeats with the following technical replicates: CXN‐mCherry = 22, mRFP‐HDEL = 41 and mRFP‐AtLNP2 = 39. Statistical analysis was performed using MANOVA followed by ANOVA with Bonferroni correction and *post hoc* Tukey honestly significant difference (HSD) tests. *Post hoc* Tukey HSD results are shown by asterisks above graphs, where * signifies a *P*‐value of 0.05–0.01, ** signifies a *P*‐value of 0.01–0.001, and *** signifies a *P*‐value of ≤0.001. (g–i) Three structural models of cisternae present in cells expressing CXN‐mCherry, mRFP‐HDEL and mRFP‐AtLNP2. (g, h) Cisternae present in cells expressing CXN‐mCherry and mRFP‐HDEL had a small distribution of small holes throughout the cisternae, whereas (i) cisternae induced by mRFP‐AtLNP2 OE had minimal or no gaps at all within the formed cisternae. Bars, 5 μm.

Although AtLNP2 OE did not initially appear to produce cisternae with a modified lumen, we decided to investigate the subresolution characteristics of the AtLNP2‐induced cisternae using GLCM analysis. GLCM is capable of uncovering changes in cisternae structure that were not immediately visible to the naked eye, using instead the underlying image texture to identify changes in cisternae structure. This analysis was applied to the distribution of the following fluorophores: GFP‐HDEL throughout the lumen of the ER in two control conditions, CXN‐mCherry (ER membrane) and mRFP‐HDEL (lumen) OE (Fig. 3a,b) and on AtLNP2 OE (Fig. 3c). The GLCM properties (i.e. contrast, correlation, energy and homogeneity, Table S3) were calculated. The measured GLCM properties showed significant differences (MANOVA: Pillai's trace, *P*‐value = 4.40 × 10^−3^ and Roy's largest root *P*‐value = 9.25 × 10^−4^) between control cisternae of both CXN‐mCherry and mRFP‐HDEL compared to mRFP‐AtLNP2 when considering cisternal energy (Fig. 3d) and homogeneity (Fig. 3e) and between mRFP‐HDEL and mRFP‐AtLNP2 for cisternal contrast (Fig. 3f; Tables S4, S5). Upon AtLNP2 OE, GFP‐HDEL distribution gave a GLCM with properties significantly closer to that expected of a consistent image texture compared to the HDEL and CXN controls. This indicated that upon mRFP‐AtLNP2 expression, the distribution of the lumenal contents was evenly spread throughout the cisternae. This would be expected in cisternae with a sac‐like structure and with a limited presence of nanoholes or tubular matrices.

The variations in image texture present in control conditions are indicative of small nanoholes being present within the cisternae (Fig. 3g,h). These nanoholes are expected in normal cisternae and have been reported across mammalian cells (Schroeder *et al*., 2019; Fuentes *et al*., 2023). What was more surprising, however, was the significant reduction in the presence of nanoholes in AtLNP2‐induced cisternae (Fig. 3i). Therefore, the cisternae induced by AtLNP2 were structurally distinct from those present in wild‐type cells.

Taken together these results suggested that the cisternae‐induced by AtLNP1 are matrix‐based tubular structures as described in mammalian cells (Nixon‐Abell *et al*., 2016), whereas AtLNP2 forms sac‐like cisternae. These two subtypes of cisternae have been reported to co‐exist in mammalian cells in differing proportions (Nixon‐Abell *et al*., 2016). AtLNP1 was able to form dense tubular matrices that could be distinguished at the edge of cisternae. The exclusion of the lumenal marker from the centre of the cisternae indicated membrane constriction preventing the flow of lumenal protein contents through the cisternae. This could either be due to a narrowed lumenal space, with AtLNP1 acting as a functional homologue for Climp‐63 in plants despite no sequence similarity (Brandizzi, 2021), or because AtLNP1 formed cisternae from dense tubular matrices, supported by limited presence in lumenal content in cisternae and the presence of dense tubular nets observable around the edges of induced cisternae. AtLNP2, however, may form sac‐like cisternal structures, consisting of two smooth expanded sections of membrane. This allows for the even distribution of GFP‐HDEL through the lumen of the cisternae.

### The AtLNP proteins do not act as lumenal spacers

One explanation for the limited lumenal content in AtLNP1‐induced cisternae is that it may act as a lumenal spacer in the same way as Climp‐63. However, in order to dimerise across the lumenal space, the majority of the protein would need to reside in the ER lumen. The alternative model, whereby AtLNP1 forms dense tubular networks, would not require a specific membrane topology. Initial predictions using TMHMM 2.0 (Krogh *et al*., 2001; Kahsay *et al*., 2005) suggested that AtLNP1 and AtLNP2 both contain two transmembrane domains near the N‐terminal with both ends of the proteins residing in the cytosol (Fig. S5). To experimentally test this prediction, we used redox‐sensitive GFP (roGFP2), a noninvasive method to test membrane topology *in planta*.

roGFP2 has modified cysteine residues which become reduced or oxidised according to the redox state of the specific cellular environment where the protein is located, causing a difference in its excitation spectra on either state (Hanson *et al*., 2004). roGFP2 has a higher excitation efficiency at 488 nm when it is found in a reducing environment, such as the cytosol, and instead a higher relative excitation efficiency at 405 nm when present in an oxidizing environment, such as the ER lumen (Brach *et al*., 2009). Therefore, ratiometric analysis of the excitation at 405 nm and 488 nm can be used to determine the presence of roGFP2 in the cytosol or ER lumen (Brach *et al*., 2009).

**Fig. 4:**
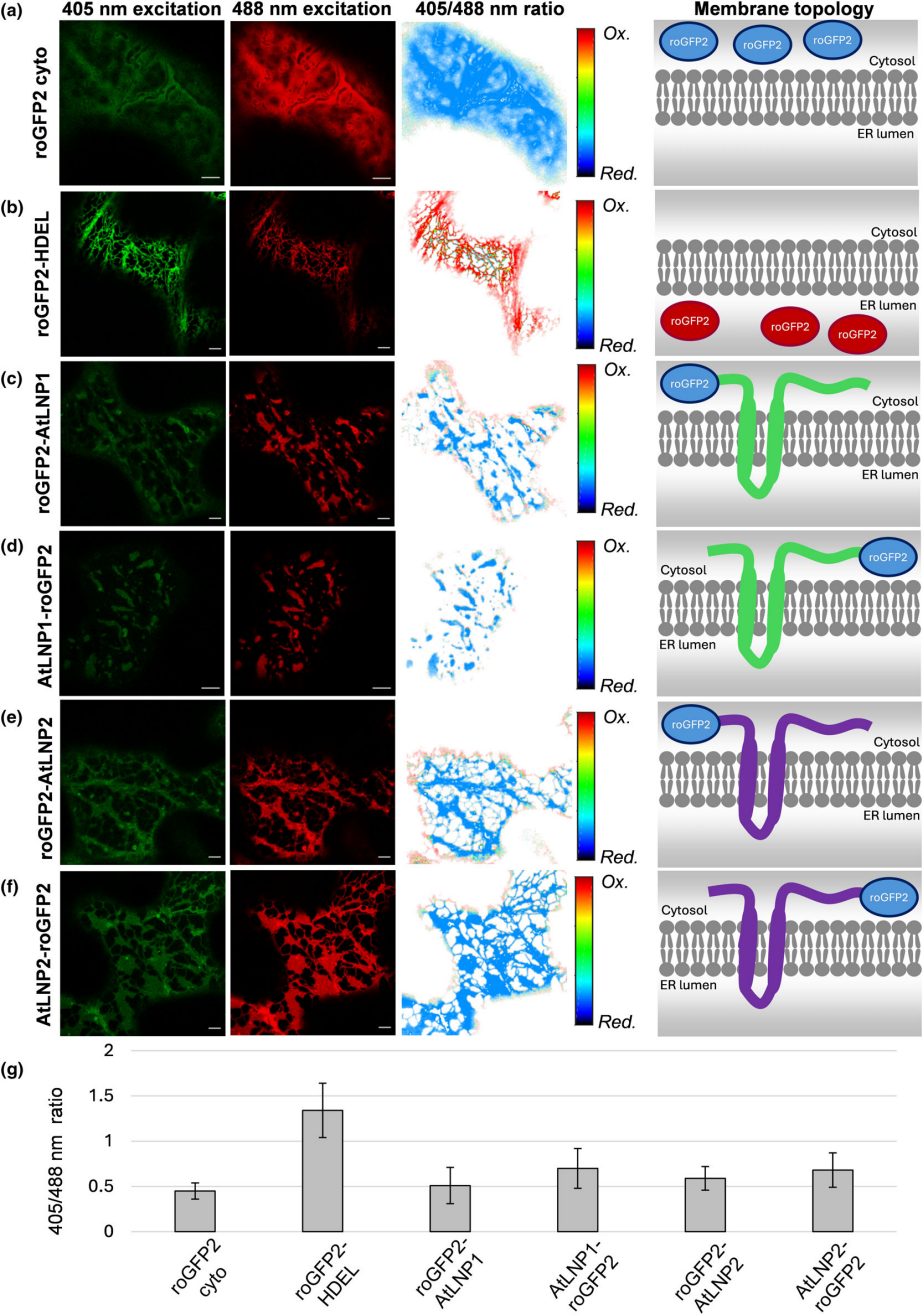
Both termini of AtLNP1 and AtLNP2 reside in the cytosol. Redox‐sensitive GFP (roGFP2) analysis of the membrane topology of AtLNP1 and AtLNP2. Representative confocal images of *Nicotiana tabacum* leaf epidermal cells expressing (a) roGFP2 cyto, (b) roGFP2‐HDEL, (c) roGFP2‐AtLNP1, (d) AtLNP1‐roGFP2, (e) roGFP2‐AtLNP2 and (f) AtLNP2‐roGFP2 after excitation at 405 nm and at 488 nm, with emission measured at 500–555 nm (left columns). The third column from the left shows a pseudocoloured ratio of the intensity of roGFP2 excitation under 405 nm vs 488 nm is shown for each image pair, where red indicates an oxidising environment (endoplasmic reticulum (ER) lumen), and blue indicates a reducing environment (cytosol). The right column shows a schematic representation of the membrane topology of each roGFP2 construct, based on the excitation ratio data. Bars, 5 μm. (g) Bar graph showing the mean excitation ratio of each construct, with SD error bars. Results taken from three biological replicates, with *n* = 10 cells per replicate per construct.

Fusion constructs of AtLNP1 and AtLNP2 with roGFP2 fused to both N‐ and C‐termini were transiently expressed in tobacco leaf epidermal cells and imaged with confocal microscopy. The ratio of roGFP2 intensity, captured across the same emission wavelengths (500–555  nm) but after excitation with the 405 nm and 488 nm lasers, was calculated on a pixel‐by‐pixel basis across each image after an initial background subtraction and bleed‐through correction (Fricker, 2016). Representative images showed that roGFP2 cyto, a cytoplasmic control, had a higher excitation at 488 nm (intensity ratio: 0.45 ± 0.09; Fig. 4a,g), while the ER lumen control showed a higher excitation at 405 nm (intensity ratio: 1.34 ± 0.30; Fig. 4b,g). Like roGFP2 cyto, both N‐ and C‐terminal AtLNP1 and AtLNP2 with roGFP2 fusions had a higher excitation at 488 nm (Fig. 4c–g; intensity ratio of roGFP2‐AtLNP1: 0.51 ± 0.20; AtLNP1‐roGFP2: 0.70 ± 0.22; roGFP2‐AtLNP2, 0.59 ± 0.13; AtLNP2‐roGFP2: 0.68 ± 0.19). These results showed that both termini of AtLNP1 and both termini of AtLNP2 reside in the cytosol.

Taking into account the topology prediction data and the results of the roGFP2 topology analysis, plant AtLNP proteins are unlikely to act as functional Climp‐63 homologues; very little of AtLNP1/2 resides in the ER lumen. This is despite AtLNPs and Climp‐63 both inducing cisternae proliferation on OE (Schroeder *et al*., 2018; Gao *et al*., 2019).

### Modified cisternae substructures impair the function of the plant secretory pathway

**Fig. 5:**
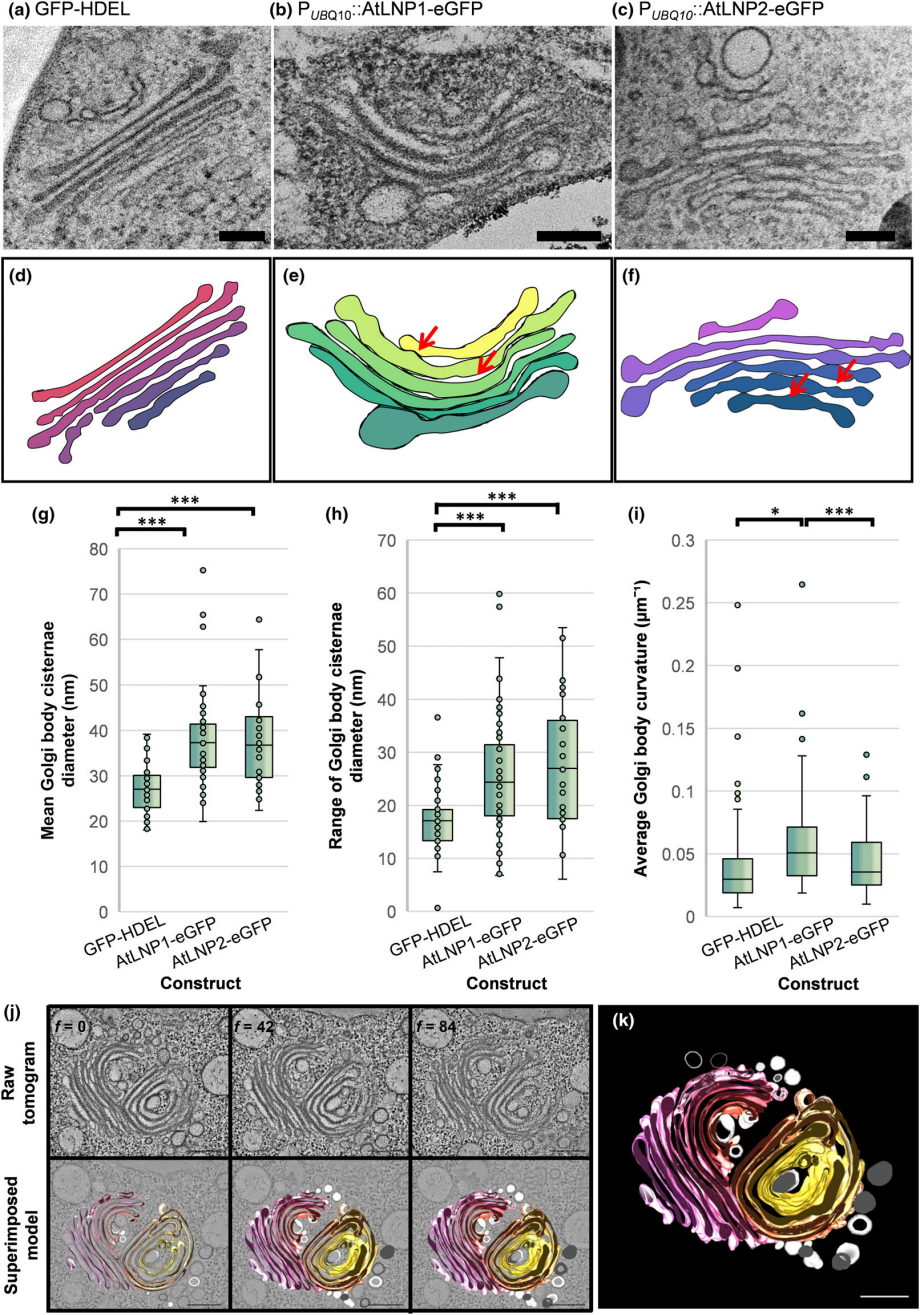
The overexpression (OE) of AtLNP1 and AtLNP2 results in curved Golgi bodies with swollen cisternae. Transmission electron microscopy (TEM) examination of Golgi body ultrastructure in cryo‐fixed *Arabidopsis thaliana* root tip cells. Representative images of Golgi bodies in Arabidopsis roots stably expressing (a) GFP‐HDEL, (b) *P*_*UBQ10*_::AtLNP1‐eGFP and (c) *P*_*UBQ10*_::AtLNP2‐eGFP alongside a tracing of the Golgi bodies for each image (d–f), showing irregular swellings along the Golgi body cisternae indicated by red arrows. Results comparing (g) the mean Golgi body cisternae diameter and (h) the range of Golgi body cisternae diameters. (i) Golgi body cisternae curvature measured by the Kappa plugin. Boxplots show median (centre line), interquartile range (box) and whiskers extending to 1.5× interquartile range; outliers are plotted as individual points. Analysis performed on GFP‐HDEL = 8, AtLNP1‐eGFP = 18, AtLNP2‐eGFP = 13 Golgi bodies. Significant differences between groups calculated using the Dunn test, where * signifies a *P*‐value of 0.05–0.01, and *** signifies a *P*‐value of ≤0.001. (j) A tomogram of two Golgi bodies in Arabidopsis overexpressing AtLNP1‐eGFP, showing both the raw data (upper panels) and the 3D reconstruction of the Golgi bodies (lower panels). (k) 3D reconstruction of a representative Golgi body on AtLNP1‐eGFP OE captured by electron tomography, showing a complex ultrastructure of curved Golgi cisternae with multiple regions of swelling. Bars, 200 nm.

The structure of the ER has previously been linked to the potential secretory capacity of cells, with an increased cisternal area associated with increased biosynthetic requirements (Stephenson & Hawes, 1986). Therefore, we decided to investigate further the impact of modified cisternae structure on the plant secretory machinery. While control Golgi bodies in GFP‐HDEL expressing roots showed the typical Golgi body structure (Fig. 5a,d), Golgi bodies in AtLNP1‐eGFP and AtLNP2‐eGFP roots had altered morphologies (Fig. 5b,c). Both AtLNP1‐eGFP and AtLNP2‐eGFP plants had Golgi body cisternae with irregular swellings along their length (red arrows, Fig. 5e,f). The mean Golgi body cisternae diameter was significantly larger in AtLNP1‐eGFP‐ and AtLNP2‐eGFP‐expressing cells, with 37.5 nm and 37.7 nm, respectively, compared to a mean of 27.2 nm on GFP‐HDEL plants (Kruskal–Wallis, *P*‐value = 5.30 × 10^−9^; Fig. 5g). In addition, AtLNP1‐eGFP and AtLNP2‐eGFP plants also exhibited larger Golgi body cisternal diameter ranges of 25.2 ± 1.47 nm and 27.5 ± 2.12 nm, respectively, compared to 17 ± 0.97 nm for GFP‐HDEL (Kruskal–Wallis, *P*‐value = 1.94 × 10^−5^, Fig. 5h). AtLNP1‐eGFP OE results in a significant increase in Golgi body curvature (Kruskal–Wallis, *P*‐value = 1.30 × 10^−5^, Fig. 5i). In some cases, this curvature was so pronounced that Golgi bodies would fold back on themselves forming a cup‐like structure (Fig. S6).

The observed swellings and increased Golgi body curvature also made it challenging to identify whether stacks within the Golgi body were in fact merged, suggesting a direct impact on Golgi body morphology. Electron tomography revealed that though the stacks may be closely appressed to each other, they did not appear to share lumenal contents and were not physically linked (Fig. 5j,k, Movie S1).

**Fig. 6:**
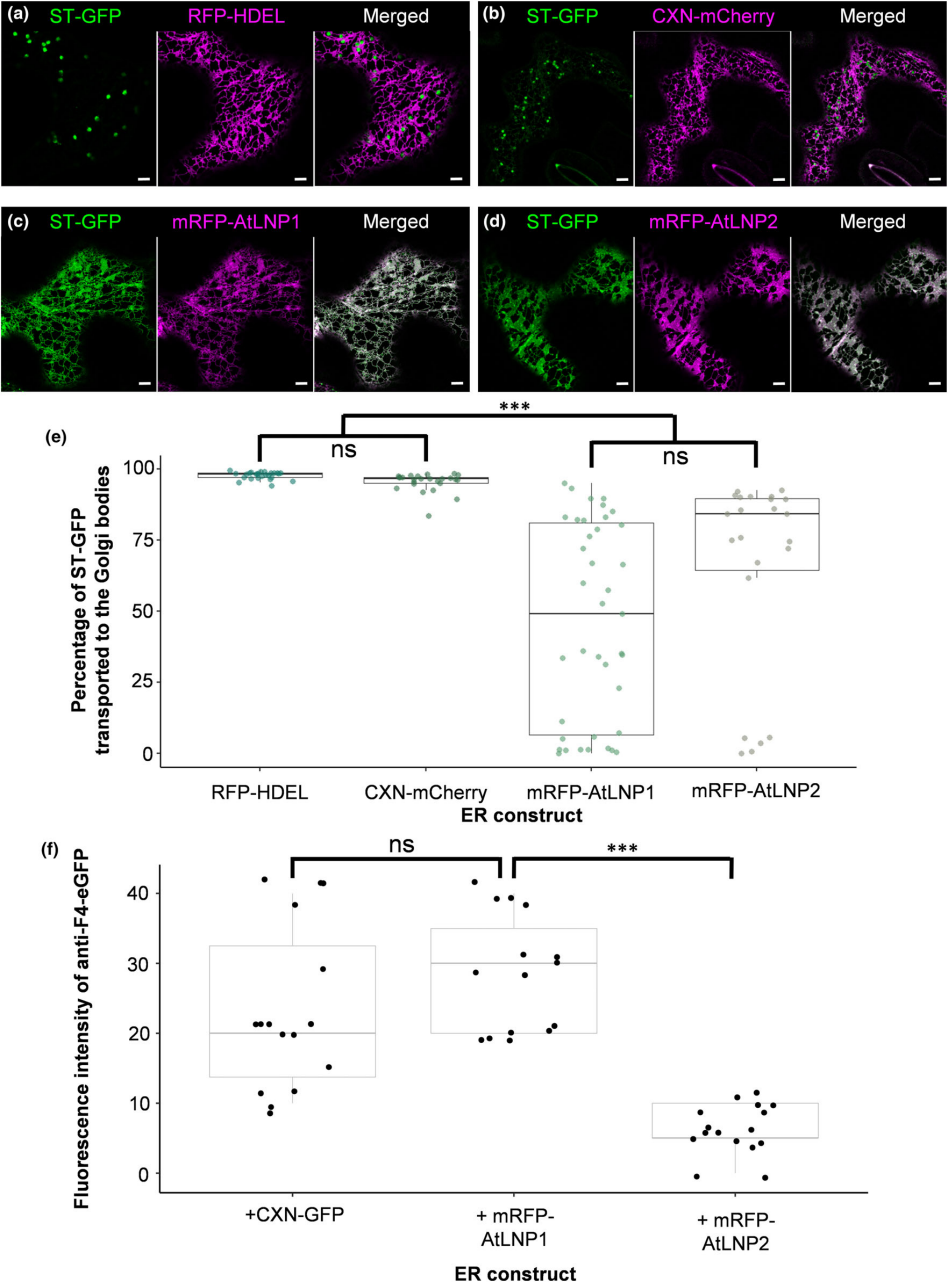
Overexpression of AtLNP1 and AtLNP2 results in impaired endoplasmic reticulum (ER)–Golgi body transport and differences in protein production capacity. Confocal microscopy analysis of the localisation of a Golgi body marker upon AtLNP protein expression. Representative images of the Golgi body marker ST‐GFP (green) alongside (a) an ER lumen control, mRFP‐HDEL; (b) an ER membrane control, CXN‐mCherry; (c) mRFP‐AtLNP1 and (d) mRFP‐AtLNP2 (all magenta). (e) A bar plot of percentage of ER to Golgi transport of two ER controls, mRFP‐AtLNP1 and mRFP‐AtLNP2 overexpression. Data were analysed using the Dunn test (ns, *P* > 0.05; ***, *P* ≤ 0.001), and error bars represent SE. Bars, 2 μm. (f) Fluorescence intensity analysis for antiF4 protein production in altered ER structural backgrounds (CXN‐GFP, AtLNP1 or AtLNP2, respectively) shows a significant decrease in antiF4 protein amount in an AtLNP2 background but a tendency to increase protein levels with AtLNP1. *n* = 6 biological repeats with at least 15 technical replicates. Standard errors are indicated, *, *P* ≤ 0.1, ***, *P* ≤ 0.001.

This altered Golgi body structure is associated with impaired ER–Golgi transport according to evidence from studies using fungal metabolites (Lippincott‐Schwartz, 1993; Satiat‐Jeunemaitre *et al*., 1996) and pharmacological agents (Tartakoff & Vassalli, 1977; Tartakoff *et al*., 1978) reducing ER–Golgi transport. Therefore, using a transient expression system, we sought to investigate whether transport between ER and Golgi bodies is impaired on AtLNP1 and AtLNP2 OE. For this, mRFP‐AtLNP1 and mRFP‐AtLNP2 were co‐expressed with the Golgi body marker sialyl transferase (ST)‐GFP which has been shown to rapidly cycle between the ER and Golgi bodies (Brandizzi *et al*., 2002). The localisation of ST‐GFP was quantified by comparing the detectable fluorescence in the ER and Golgi bodies and calculating the percentage fluorescence intensity present in the Golgi bodies. Higher values represented normal ER to Golgi body transport whereas lower values signified that ST‐GFP was not transported from the ER to the Golgi bodies. mRFP‐HDEL and CXN‐mCherry were used as controls for OE as nonfunctional lumenal and membrane proteins, respectively. No significant difference in ER–Golgi body transport was identified on OE of mRFP‐HDEL vs CXN‐mCherry (Fig. 6a,b), however, OE of mRFP‐AtLNP1 and 2 (Fig. 6c,d) resulted in impaired ER–Golgi body transport (Kruskal–Wallis, *P*‐value 2.2 × 10^−16^, Fig. 6e). ST‐GFP did not become associated with swollen Golgi body‐associated vesicles, nor was it entirely retained within the Golgi body itself. This suggests that the changes to Golgi body morphology were more likely to be due to impaired ER–Golgi body transport than inhibited protein transport through the Golgi body itself.

In addition, protein production in the different AtLNP backgrounds was investigated (Fig. 6f). For this, the high‐value recombinant protein antiF4, an antibody against enterotoxigenic *Escherichia coli* bacteria, that has been shown to protect against disease when supplemented into animal feed (Virdi *et al*., 2013), was fused to the ER targeting sequence of sporamin and the ER‐retention signal HDEL to achieve ER localisation. Fluorescence intensity of antiF4‐eGFP was measured in tobacco epidermal cells as a proxy for protein amount. Tobacco cells overexpressing mRFP‐AtLNP1 showed no statistically significant difference in antiF4 production compared to tobacco cells expressing the ER membrane marker CXN‐GFP but a trend to increased antiF4 levels. However, AtLNP2 OE resulted in statistically decreased antiF4 levels indicating that the cisternal substructure in AtLNP1‐ and AtLNP2‐induced cisternae had different effects on (recombinant) protein expression. These results were further validated by Western Blotting antiF4‐eGFP with anti‐GFP on tobacco epidermal leaves overexpressing either CXN‐GFP, mRFP‐AtLNP1 or mRFP‐AtLNP2, respectively. Overexpression of mRFP‐AtLNP1 results in a significant increase in the antiF4‐eGFP production, while mRFP‐AtLNP2 OE results in a significant decrease in antiF4 production (Fig. S7).

Impaired ER–Golgi body transport and altered Golgi structure by AtLNP1 and AtLNP2 OE is expected to block protein secretion to the apoplast, as transport from the ER to Golgi bodies is an essential step in the secretory pathway. SP‐mCherry, a default secretory pathway marker (da Costa *et al*., 2010), was secreted to the apoplast when co‐expressed with GFP‐HDEL and CXN‐GFP (Fig. S8a,b) but appeared largely retained in the ER on OE of AtLNP1‐eGFP and AtLNP2‐eGFP (Fig. S8c–e, Kruskal–Wallis, *P*‐value = 5.85 × 10^−14^), as expected from the impaired ER to Golgi body transport.

These data indicate that ER structure modifications via AtLNP proteins (and potentially other ER morphogens) impact upon ER functionality, as shown here for ER–Golgi transport, the cellular secretory pathway and protein production. Interestingly, though protein transport and secretion appear to be impaired, root length assays show an increase in mean root length on OE of AtLNP1‐eGFP in Arabidopsis stable lines (Fig. S9).

## Discussion

### The role of AtLNP1 and AtLNP2 in forming cisternae with distinct morphologies

The morphology of the ER is highly dynamic, regulated by specialized proteins known as ER shapers, such as AtLNP1 and AtLNP2, which play crucial roles in the formation of structurally distinct cisternae. AtLNP1 generates cisternae with dense tubular matrices and compressed lumens, akin to structures observed in mammalian cells (Shemesh *et al*., 2014). AtLNP1 is believed to stabilise negative membrane curvature, facilitating the creation of cisternae with tubular matrices (Wang *et al*., 2018). On the other hand, AtLNP2 forms cisternae with more contiguous lumen, presenting a more typical sac‐like structure. Thus, the two proteins may contribute to the formation of different cisternae with distinct functional roles. The cisternae formed by AtLNP2 may support metabolon formation and metabolite storage by providing a large continuous surface area, while those created by AtLNP1 may serve as reservoirs for membrane proteins and lipids, potentially aiding rapid ER remodelling during stress, such as during fungal infection (Xie *et al*., 2019).

### Climp‐63 and Arabidopsis LNP proteins have related functions but most likely different mechanistic properties

In contrast to AtLNP1 and AtLNP2, the mammalian protein Climp‐63 also induces cisternae formation but operates through a fundamentally different mechanism. Climp‐63 is an intralumenal spacer that dimerises across the ER lumen, whereas both termini of AtLNP1 and AtLNP2 are cytosolic, suggesting that their mechanism of action differs (Shibata *et al*., 2010; Shen *et al*., 2019). Notably, no Climp‐63 homologue has been identified in plants, indicating that AtLNP proteins, particularly AtLNP2, may have diversified functions to fulfil this role (Shen *et al*., 2019). An important consideration is whether the intralumenal domains of AtLNPs could act as spacers or adhesive elements across the cisternal lumen. Based on both topology predictions and our roGFP2 analysis, the lumen‐exposed regions of AtLNP1 and AtLNP2 are extremely short, making it unlikely that they could function as classical lumenal spacers analogous to Climp‐63, which forms extended lumenal coiled‐coil dimers to maintain sheet spacing (Shibata *et al*., 2010; Shen *et al*., 2019). Indeed, the majority of each AtLNP protein resides on the cytosolic face of the ER membrane (Brach *et al*., 2009). An alternative possibility is that these short lumenal segments could mediate transient ‘press‐stud’–like interactions between opposing membranes, locally tethering them and generating a nanohole‐rich cisternal texture rather than acting as true spacers. Such nanoholes have been described in ER cisternae in mammalian cells and are thought to contribute to cisternal plasticity (Schroeder *et al*., 2019; Fuentes *et al*., 2023). However, our data argue against this being the dominant mechanism underlying AtLNP1‐induced cisternae. The pronounced exclusion of lumenal GFP‐HDEL from the cisternal centre, together with the frequent appearance of dense tubular matrices at cisternal edges, is more consistent with cisternae composed of tightly packed or highly branched tubules, as described for dense tubular matrices in mammalian ER, rather than apposed membranes linked by lumenal adhesion (Nixon‐Abell *et al*., 2016). We therefore favour a model in which AtLNP1 primarily promotes negative membrane curvature and tubular network formation, with apparent lumenal restriction arising as a secondary consequence of dense tubule packing rather than direct lumenal tethering.

### AtLNP1 and AtLNP2 affect ER–Golgi body transport and overall protein secretion

AtLNP1 and AtLNP2 are implicated in altering ER–Golgi body transport. Overexpression of AtLNP1 or AtLNP2 results in swollen and curved Golgi cisternae, reminiscent of phenotypes observed in Brefeldin A (BFA) very‐low‐density lipoprotein‐treated plant cells, which impair ER–Golgi transport (Saint‐Jore *et al*., 2002). This suggests that AtLNP1 and AtLNP2 may disrupt the anterograde transport between the ER and Golgi, a crucial process for proper protein secretion (Lippincott‐Schwartz, 1993). Research on human cells has shown that morphogen imbalances can hinder ER–Golgi transport, such as in the case of reduced RTN3 expression, which impairs very‐low‐density lipoprotein transport in liver cells (Siddiqi *et al*., 2018). Likewise, in HeLa cells, reduced RTN4a expression decreases surface trafficking of secreted proteins (Mukherjee & Levy, 2019). These findings suggest that a correct balance of ER morphogens, including AtLNP1 and AtLNP2, is essential to maintain proper protein flux between the ER and Golgi.

Interestingly, AtLNP1 OE appears to enhance protein production, correlating with increased auxin biosynthetic enzyme levels and subsequent auxin synthesis (Kriechbaumer & Brandizzi, 2020). By contrast, AtLNP2 OE impairs protein production, suggesting that the balance of these proteins plays a significant role in regulating protein synthesis in the ER. This has important implications for the production of high‐value proteins in plants, such as antibodies against enterotoxigenic *E. coli*, which have potential as supplements in animal feed to protect against disease (Virdi *et al*., 2013). Plant systems for recombinant protein production are rapidly growing, and optimising ER structure to increase protein yield could significantly impact this industry (Twyman *et al*., 2005; Moon *et al*., 2019; Ghag *et al*., 2021).

**Fig. 7:**
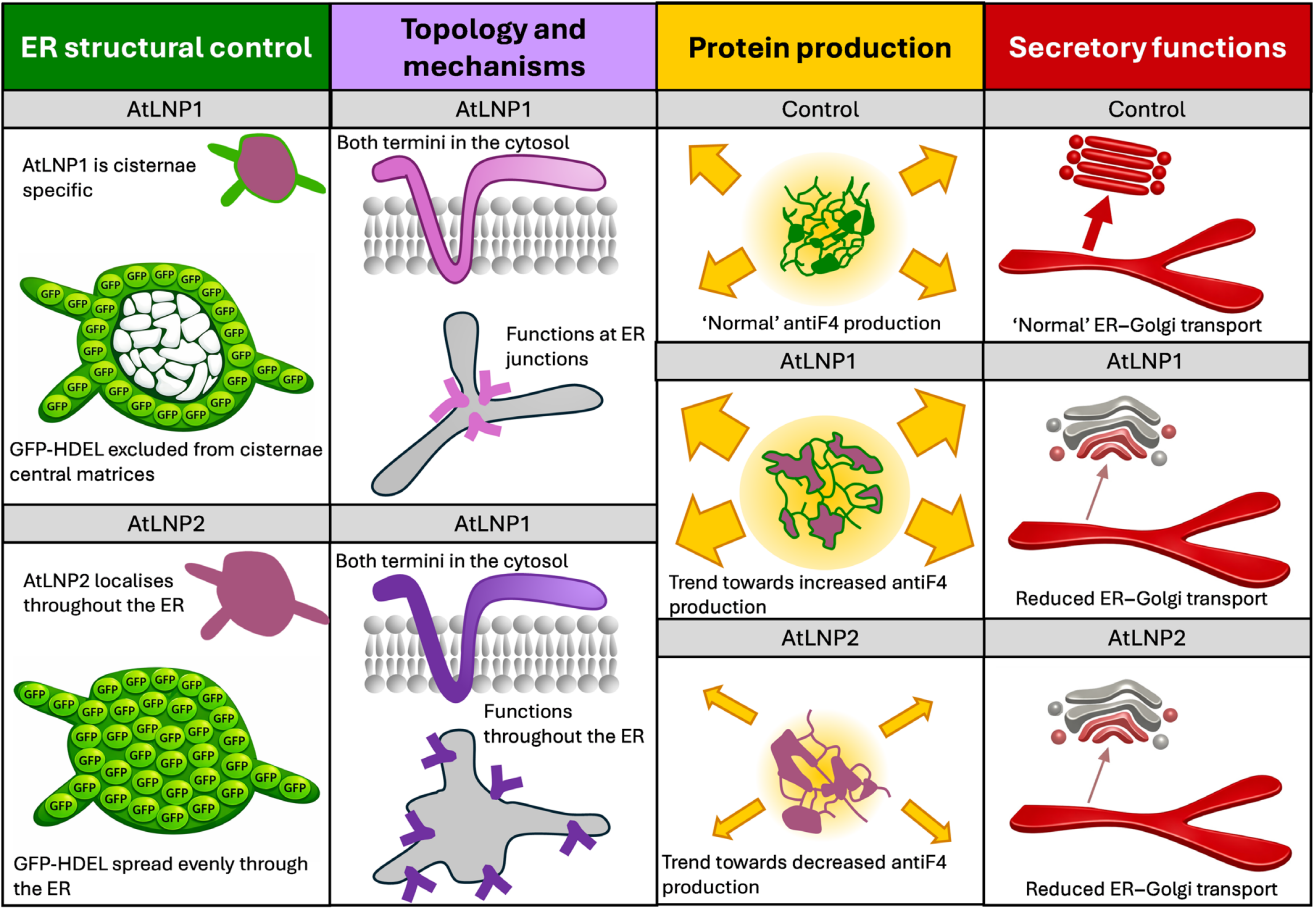
Model for the role of AtLNPs in endoplasmic reticulum (ER) structure and function. This model integrates four major conclusions from our study, each color‐coded: ER structural organization (green), protein topology and mechanism (purple), impacts on recombinant protein production (orange), and ER–Golgi transport (red). AtLNP1 and AtLNP2 differentially shape ER architecture. AtLNP1 drives the formation of distinctive cisternae with a lumen that excludes GFP‐HDEL, whereas AtLNP2 is distributed throughout the ER and generates cisternae with canonical luminal accessibility. The results of roGFP2 analysis indicate both AtLNPs possess cytosolic N‐ and C‐termini. Mechanistically, AtLNP1 may promote negative membrane curvature at small junctions, contributing to a highly reticulated cisternal network, whereas AtLNP2 acts broadly across the ER without concentrating at discrete curvature sites. Overexpression of AtLNP1 is associated with a trend towards enhanced production of certain recombinant proteins (e.g. antiF4), an effect not seen with AtLNP2 overexpression. Finally, perturbation of either AtLNP alters ER–Golgi transport dynamics.

In conclusion, we propose that AtLNP1 and AtLNP2 play complementary roles in the formation of structurally distinct cisternae (Fig. 7). AtLNP1 likely initiates the aggregation of ER membranes into dense tubular matrices, which are then stabilised through negative curvature and membrane junctions (Sun *et al*., 2020). AtLNP2, on the other hand, may convert these tubular matrices into more typical sheet‐like cisternae with contiguous lumens. The balance between these two proteins is critical for maintaining proper ER–Golgi transport and efficient protein secretion. AtLNP1 and AtLNP2 may also represent two stages in cisternal formation, with AtLNP1 serving as an initial aggregate stabiliser and AtLNP2 expanding these structures into stable cisternae. Alternatively, they may function locally and separately, each forming cisternae with different structures and distinct, complementary functions. Understanding how these proteins modulate ER structure and function could provide valuable insights into optimising plant systems for the production of high‐value proteins.

## Competing interests

None declared.

## Author contributions

CP, VK, FB and SWB conceived of the experiments. CP, NF, TSR, CM, FM‐L, AC and VK carried out the experiments. CP, NF, CM, MWA and VK performed the data analysis. The manuscript was written by CP, FB and VK. All authors contributed to the article and approved the submitted version.

## Disclaimer

The New Phytologist Foundation remains neutral with regard to jurisdictional claims in maps and in any institutional affiliations.

## Supporting information


**Fig. S1** Example outputs of ImageJ plugins used to analyse Golgi body cisternae structure.
**Fig. S2** Characterisation of LNP expression transient (tobacco epidermal leaf cells) and stable (Arabidopsis) systems.
**Fig. S3** Analysis of the lumenal characteristics of cisternae in AtLNP1 and AtLNP2 co‐expression.
**Fig. S4** EM tomography of ER cisternae in Arabidopsis root cells.
**Fig. S5** The membrane topology of AtLNP1 and 2 predicted by TMHMM 2.0.
**Fig. S6** Cup‐shaped Golgi bodies observed in Arabidopsis roots stably expressing AtLNP1‐eGFP.
**Fig. S7** Characterisation of transient antiF4 production in different ER structural backgrounds (CXN‐GFP, AtLNP1, AtLNP2) in tobacco epidermal leaf cells.
**Fig. S8** AtLNP1 and 2 overexpression results in blocked transport to the apoplast.
**Fig. S9** Comparison of mean root length of Arabidopsis lines after 10 d of growth.
**Table S1** Constructs and stable lines used/generated as part of this work.
**Table S2** Typical confocal laser power used throughout this work.
**Table S3** Calculation of GLCM properties.
**Table S4** Summary of statistical comparison of GLCM properties.
**Table S5** Calculation of GLCM properties.

**Video S1.** Electron tomography model of Golgi body structure on AtLNP1‐eGFP overexpression.
Please note: Wiley is not responsible for the content or functionality of any Supporting Information supplied by the authors. Any queries (other than missing material) should be directed to the *New Phytologist* Central Office.

## Data Availability

Data are available in the supplementary material (Figures S1–S9; Tables S1–S5). Accession numbers: AtLNP1 AT2G24330 and AtLNP2 AT4G31080.
