# Supplementary material for: Built different: ER cisternae formed by the Arabidopsis Lunapark proteins differ in ultrastructure and affect ER–Golgi transport
Source: New Phytol. 2026 Apr 29;250(6):3828–45. doi: 10.1111/nph.71217 (PMC13193348; doi:10.1111/nph.71217)
Supplement: Supplementary file 1 — Fig. S1 Example outputs of ImageJ plugins used to analyse Golgi body cisternae structure. Fig. S2 Characterisation of LNP expression transient (tobacco epidermal leaf cells) and stable (Arabidopsis) systems. Fig. S3 Analysis of the lumenal characteristics of cisternae in AtLNP1 and AtLNP2 co‐expression. Fig. S4 EM tomography of ER cisternae in Arabidopsis root cells. Fig. S5 The membrane topology of AtLNP1 and 2 predicted by TMHMM 2.0. Fig. S6 Cup‐shaped Golgi bodies observed in Arabidopsis roots stably expressing AtLNP1‐eGFP. Fig. S7 Characterisation of transient antiF4 production in different ER structural backgrounds (CXN‐GFP, AtLNP1, AtLNP2) in tobacco epidermal leaf cells. Fig. S8 AtLNP1 and 2 overexpression results in blocked transport to the apoplast. Fig. S9 Comparison of mean root length of Arabidopsis lines after 10 d of growth. Table S1 Constructs and stable lines used/generated as part of this work. Table S2 Typical confocal laser power used throughout this work. Table S3 Calculation of GLCM properties. Table S4 Summary of statistical comparison of GLCM properties. Table S5 Calculation of GLCM properties. [file NPH-250-3828-s001.pdf]

***New Phytologist* Supporting Information**

Article title: **Built different: ER cisternae formed by the Arabidopsis Lunapark proteins differ in ultrastructure and affect ER-Golgi transport**

Authors: Charlotte Pain[1,2], Tatiana Spatola Rossi[1,2], Nadine Field[1,2], Carmen Mata[1,2], Alessia Candeo[3], Muhammad Ali[1,2], Flavia Moreira-Leite[2], Stanley W. Botchway[4], Federica Brandizzi[5,6,7], Verena Kriechbaumer[1,2]

Article acceptance date: 27 March 2026

The following Supporting Information is available for this article:

Supplementary Table S1: Constructs and stable lines used/generated as part of this work. A list of constructs used as part of this study and the publication of origin where applicable. Stable Arabidopsis lines used as a part of this study are also listed.

**Constructs used in this study that have been previously published**	
mRFP-AtLNP1	(Pain, Kriechbaumer et al. 2019)
mRPP-AtLNP2	
AtLNP1-eGFP	(Kriechbaumer, Breeze et al. 2018)
AtLNP2-eGFP	
GFP-HDEL	(Brandizzi, Hanton et al. 2003)
mRFP-HDEL	
CXN-mCherry	(Groves, McKenna et al. 2019)
CXN-GFP	(Irons, Evans et al. 2003)
ST-GFP	(Saint-Jore, Evins et al. 2002)
roGFP2-HDEL	(Schwarzländer, Fricker et al. 2008)
roGFP2 cytosolic	(Wang, Hummel et al. 2011)
**Constructs generated as part of this study**	
roGFP2-AtLNP1	
AtLNP1-roGFP2	
roGFP2-AtLNP2	
AtLNP2-roGFP2	
antiF4-eGFP	
**Stable Arabidopsis lines used as part of this study**	
UB10:AtLNP1-eGFP	(Kriechbaumer, Breeze et al. 2018)
UB10:AtLNP2-eGFP	
35S::GFP-HDEL	(Zheng, Kunst et al. 2004)
35S::CXN-GFP	(Irons, Evans et al. 2003)

Supplementary Table S2: Typical confocal laser power used throughout this work.

Typical laser power used throughout this study alongside the fluorophore/dye used. Some small variations possible based on system warmup time and fluorophore expression.

**Experiment**	**Fluorophore**	**Laser**	**Laser power (µW)**
N. tabacum leaf transient expression	GFP	488 nm	8.9
	RFP	561 nm	56.0
roGFP topology in *N. tabacum* leaves	Channel overlap	405 nm	27.3
	roGFP2	405 nm	52.1
	roGFP2	488 nm	72.5
Arabidopsis cotyledons	Rhodamine B	514 nm	13.7

Supplementary Table S3: Calculation of GLCM properties.

Full details as to the calculation of relevant GLCM properties as performed in MATLAB.

**Statistic**	**Description**	**Equation**
Contrast	Local intensity variations detected in the GLCM	$\sum_{i,j} \lvert i-j \rvert^2 p(i, j)$
Correlation	The joint probability of specific pixel pairs	$\sum_{i, j} \frac{(i-\mu i)(j-\mu j)p(i,j)}{\sigma_i \sigma_j}$
Energy	The sum of squared elements in the GLCM	$\sum_{i,j} p(i, j)^2$
Homogeneity	How closely the distribution of the GLCM is to the diagonal	$\sum_{i, j} \frac{p(i, j)}{1 + \lvert i-j \rvert}$

Supplementary Figure S1: Example outputs of ImageJ plugins used to analyse Golgi body cisternae structure.

(a) Example output of the Kappa analysis ImageJ plugin. Cyan lines show the fitted line tracing the Golgi body cisternae. (b) Example output of the integrated distance macro, with the Golgi body cisternae edge highlighted in green and the distances measured shown by yellow lines.

a)

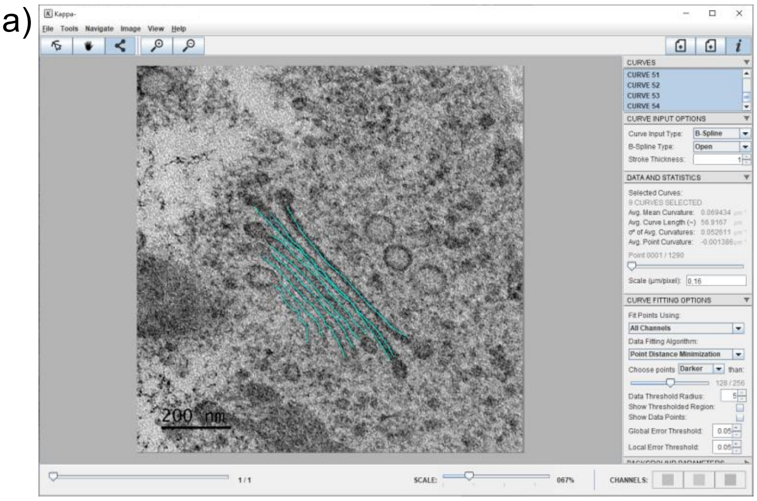

b)

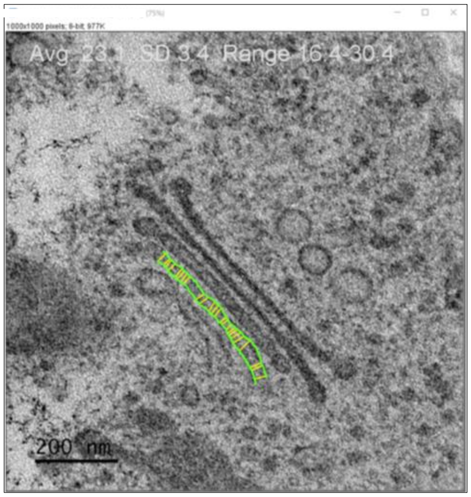

Supplementary Figure S2: Characterisation of LNP expression transient (tobacco epidermal leaf cells) and stable (Arabidopsis) systems.

RNA levels (Transcripts Per Million, TPM) were quantified using RNA-seq. Leaves infiltrated with agrobacteria (control) were compared to leaves after agrobacterium-mediated transient over-expression of a) AtLNP1 (AtLNP1 OE) or b) AtLNP2 (AtLNP2 OE), respectively. Biological replicates n=3. Endogenous NtLNP1 or 2 levels, respectively, showed no significant difference between control and OE (NtLNP1 = 59 ± 5 TPM; NtLNP2 = 48 ± 6 TPM but overall AtLNP1/2 levels increased significantly after the transient expression of AtLNP1 or 2 (Nt/AtLNP1 = 307 ± 14 TPM; Nt/AtLNP2 = 199 ± 24 TPM). *** signifies a p-value ≤ 0.001. c) RNA-seq was performed on WT Arabidopsis plants and Arabidopsis expressing AtLNP1 or AtLNP2, respectively, in a stable manner. Biological replicates n=3. WT AtLNP1 and AtLNP2 transcript levels were significantly increased in the over-expression plants (WT AtLNP1 = 16.2 ± 0.2 TPM, WT AtLNP2 = 17.4 ± 0.1 TPM, AtLNP1 OE = 18.4 ± 0.1 TPM, AtLNP2 OE = 19.5 ± 0.1 TPM.

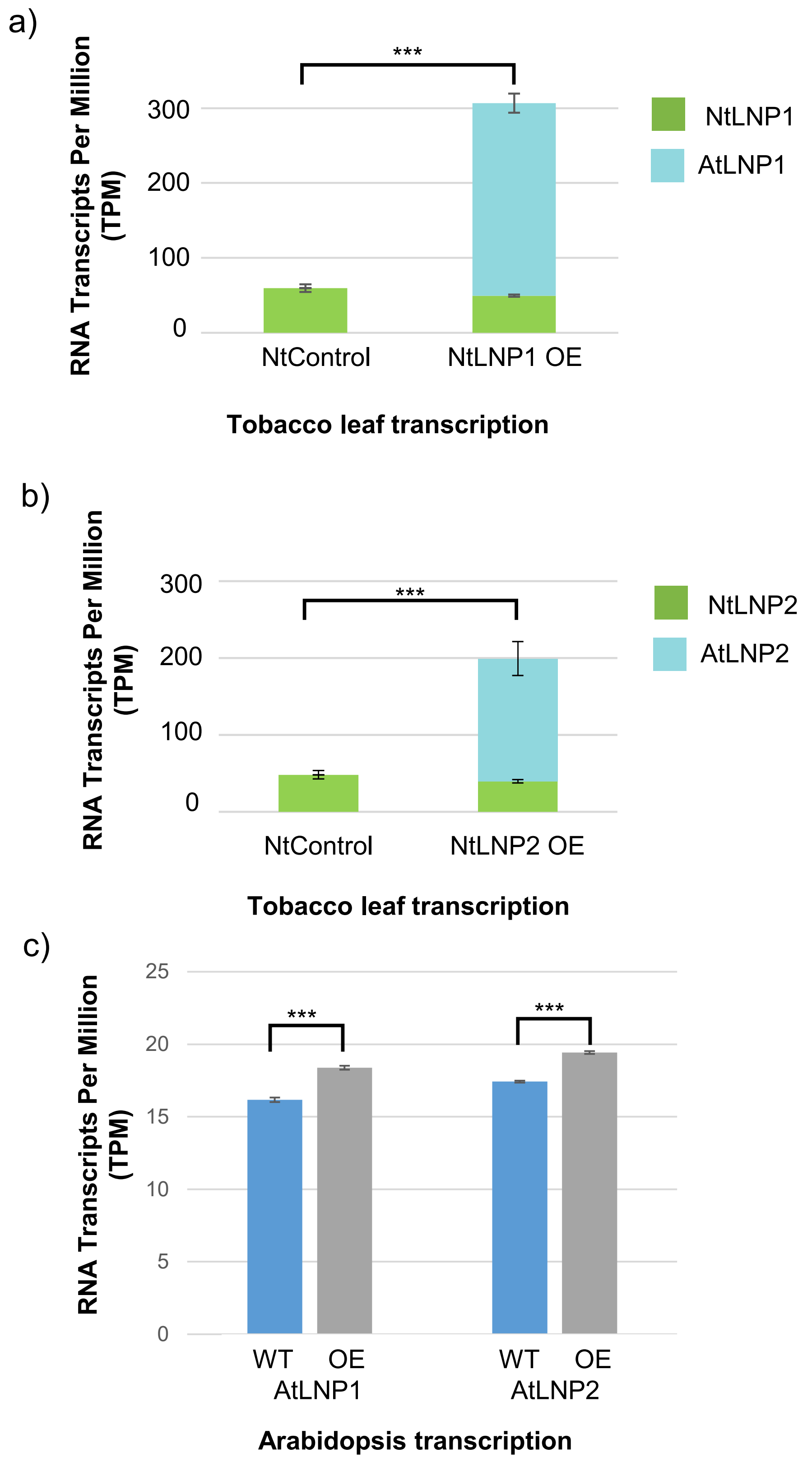
a)
***
RNA Transcripts Per Million (TPM)
300
200
100
0
NtControl
NtLNP1 OE
Tobacco leaf transcription
NtLNP1
AtLNP1
b)
***
RNA Transcripts Per Million (TPM)
300
200
100
0
NtControl
NtLNP2 OE
Tobacco leaf transcription
NtLNP2
AtLNP2
c)
***
***
RNA Transcripts Per Million (TPM)
25
20
15
10
5
0
WT
OE
AtLNP1
WT
OE
AtLNP2
Arabidopsis transcription

Supplementary Figure S3: Analysis of the lumenal characteristics of cisternae in AtLNP1 and AtLNP2 co-expression.

(**a**) Representative confocal micrograph of *N. tabacum* leaf epidermal cells expressing both AtLNP1-eGFP (green) and mRFP-AtLNP2 (magenta) at approximately equal levels co-expressed with the ER lumenal marker mTagBFP2--HDEL (blue) alongside a merged image showing all three channels. (**b**) Images are insets of area highlighted by yellow box in a), showing details of cisternal areas and their lumenal characteristics. Scale bars = 5 µm. (**c**) Radial intensity plots of AtLNP1-eGFP (green), mRFP-AtLNP2 (magenta) and mTagBFP2-HDEL (blue), with standard deviations shown around the mean result. Lack of standard deviation bars towards the right-hand side of the graph indicates an insufficient number of cisternae of that size included in the analysis to produce a standard deviation bar. Results are shown for 3 biological repeats with 8 technical replicates.

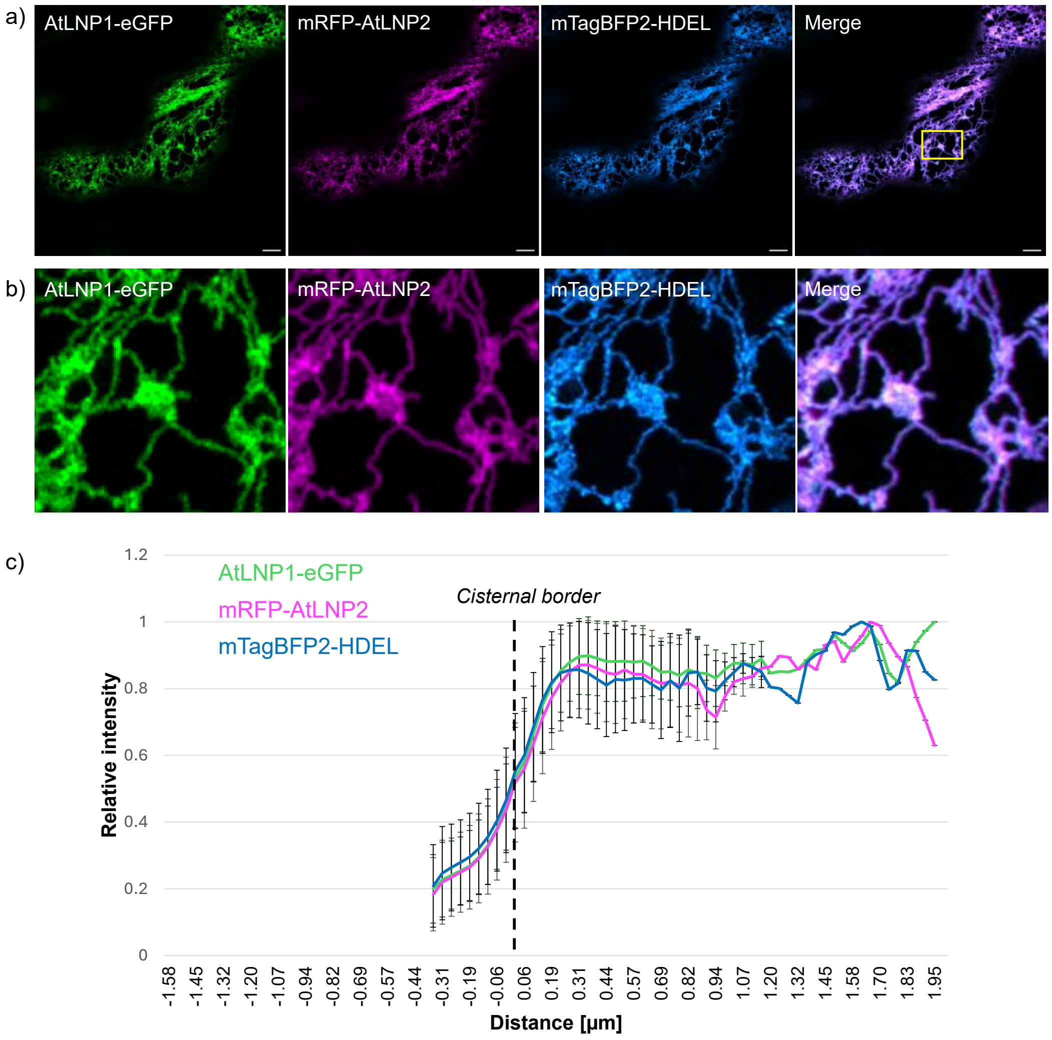

Supplementary Table S4: Summary of statistical comparison of GLCM properties.

Table showing the output of statistical comparison of GLCM properties of the GFP-HDEL fluorescence signal in cisternae marker with CXN-mCherry, mRFP-HDEL and mRFP-AtLNP2. The p-value of each ANOVA is shown after a Bonferroni correct, alongside a post-hoc Tukey HSD analysis between all the included groups. If the initial ANOVA result did not show a significant change, no post-hoc test was performed.

Property	ANOVA p-value (corrected)	Group 1	Group 2	Tukey p-value
**Cisternal contrast**	2.30×10^{-3}	CXN-mCherry	mRFP-HDEL	1
		CXN-mCherry	mRFP-AtLNP2	7.7×10^{-2}
		mRFP-HDEL	mRFP-AtLNP2	2.16×10^{-3}
**Cisternal correlation**	0.32			
**Cisternal energy**	2.77×10^{-7}	CXN-mCherry	mRFP-HDEL	1
		CXN-mCherry	mRFP-AtLNP2	9.13×10^{-3}
		mRFP-HDEL	mRFP-AtLNP2	4.36×10^{-4}
**Cisternal homogeneity**	7.06×10^{-5}	CXN-mCherry	mRFP-HDEL	1
		CXN-mCherry	mRFP-AtLNP2	3.31×10^{-3}
		mRFP-HDEL	mRFP-AtLNP2	1.35×10^{-4}

Supplementary Table S5: Calculation of GLCM properties.

Full details as to the calculation of relevant GLCM properties as performed in MATLAB.

Statistic	Description	Calculation
Contrast	Local intensity variations detected in the GLCM	$\sum_{i,j} \lvert i-j \rvert^2 p(i,j)$
Correlation	The joint probability of specific pixel pairs	$\sum_{i,j} \frac{(i-\mu j)(j-\mu j)p(i,j)}{\sigma_i \sigma_j}$
Energy	The sum of squared elements in the GLCM	$\sum_{i,j} p(i,j)^2$
Homogeneity	How closely the distribution of the GLCM is to the diagonal	$\sum_{i,j} \frac{p(i,j)}{1+\lvert i-j \rvert}$

Supplementary Figure S4: EM tomography of ER cisternae in Arabidopsis root cells.

(a) An EM tomogram of the ER network in Arabidopsis cryo-fixed root tip cells overexpressing P_{UBQ10}::AtLNP1-eGFP and (b) P_{UBQ10}::AtLNP2-eGFP, showing both the raw data of the mid-point in the tomogram (upper panels) and the lower-point with 3D reconstructions of the ER network superimposed (lower panels). Scale bars = 200 µm.

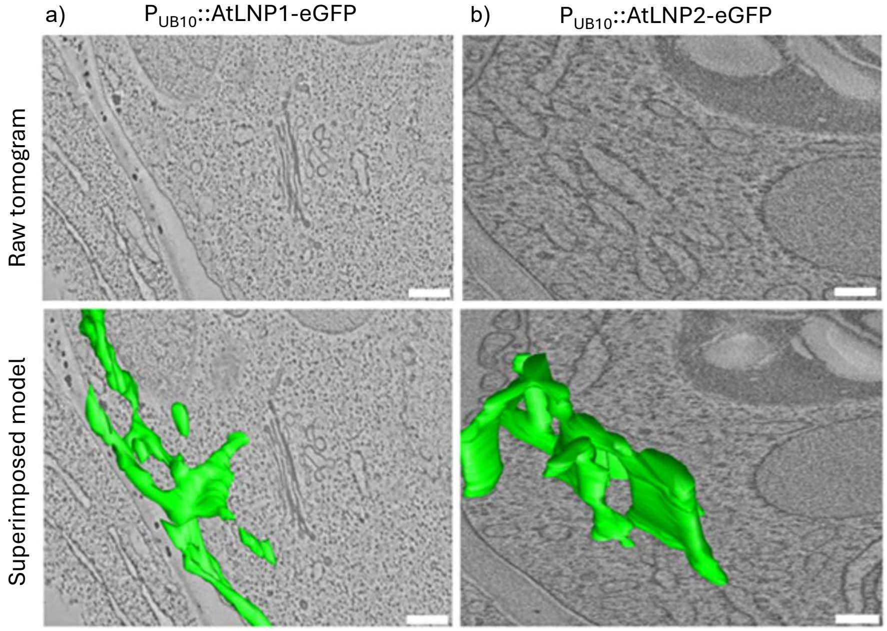

Supplementary Figure S5: The membrane topology of AtLNP1 and 2 predicted by TMHMM 2.0.

Membrane topology prediction of (a) AtLNP1 and (b) AtLNP2 created by TMHMM – 2.0. Two transmembrane domains are predicted for each protein (grey) near the N-terminal, with both termini of each protein predicted to reside cytoplasmic side of the ER (orange) and only a small segment between the transmembrane domains (blue) predicted to face the ER lumen.

a)

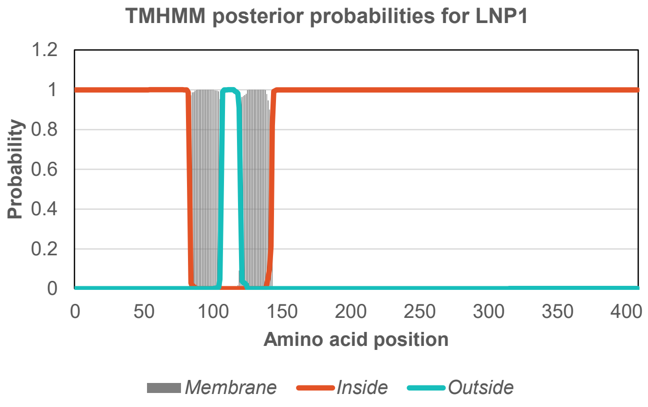

b)

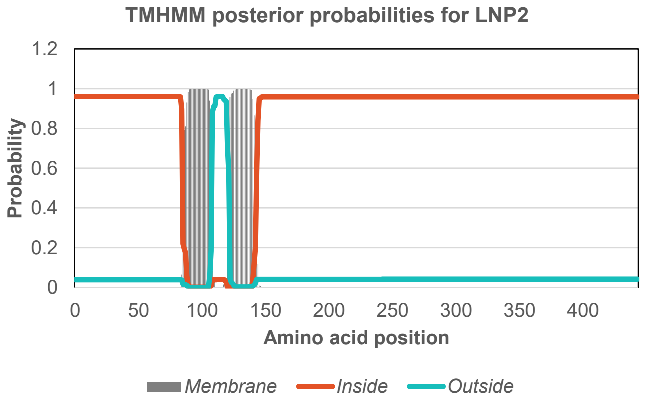

Supplementary Figure S6: Cup-shaped Golgi bodies observed in Arabidopsis roots stably expressing AtLNP1-eGFP.

Images collected using transmission electron microscopy (TEM) of high-pressure frozen tissues. Three example images of highly curved Golgi bodies that were identified in Arabidopsis plants expressing AtLNP1-eGFP.

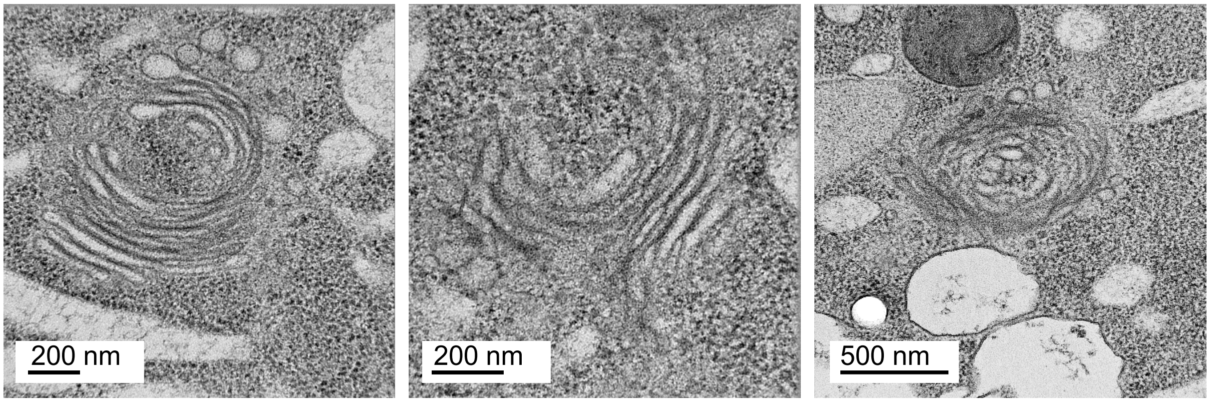

Supplementary Video/Movie S1: Electron tomography model of Golgi body structure on AtLNP1-eGFP overexpression.

Movie showing first the raw electron tomography data after alignment and then the Golgi body model superimposed on the raw data. Scale bar 200 nm.

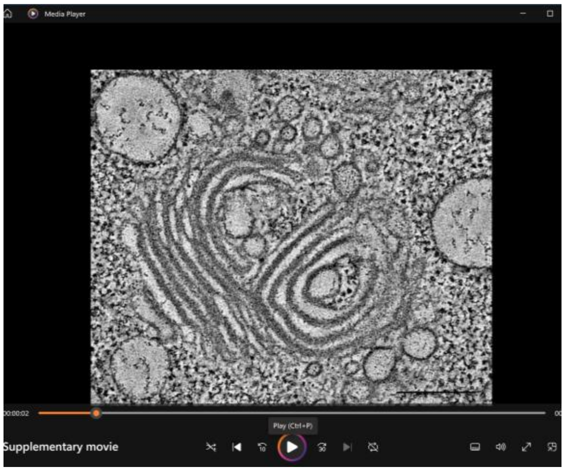

Supplementary Figure S7: Characterisation of transient antiF4 production in different ER structural backgrounds (CXN-GFP, AtLNP1, AtLNP2) in tobacco epidermal leaf cells.

a) antiF4-eGFP was transiently expressed in tobacco leaves infiltrated with the ER membrane marker Calnexin-GFP (CXN-GFP, 1) or AtLNP1 (2) or AtLNP2 (3), respectively. Western Blotting with anti-GFP antibodies was carried out and b) intensity of the antiF4-eGFP band was quantified and normalised to the control. Biological replicates n=3. * signifies a p-value = 0.05-0.01, ** signifies a p-value = 0.01-0.001. c) Bar plot representation of Fluorescence intensity analysis for antiF4 protein production in altered ER structural backgrounds (CXN-GFP, AtLNP1 or AtLNP2, respectively) shows a significant decrease in antiF4-eGFP protein amount in a AtLNP2 background but a tendency to increase protein levels with AtLNP1. n = 6 biological repeats with at least 15 technical replicates. Standard errors are indicated, * p ≤ 0.1*** p ≤ 0.001.

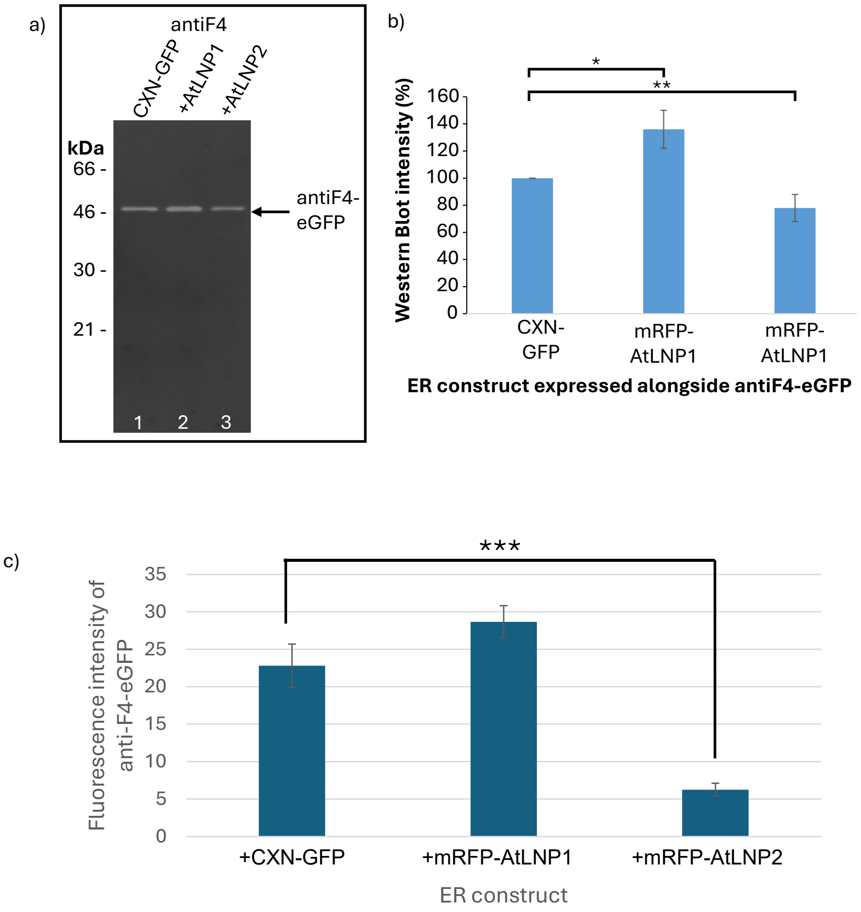

Supplementary Figure S8: AtLNP1 and 2 overexpression results in blocked transport to the apoplast.

Images collected using confocal microscopy with Airyscan showing transient overexpression of the secretory pathway marker SP-mCherry (magenta) alongside (a) GFP-HDEL, (b) CXN-GFP, (c) AtLNP1-eGFP and (d) AtLNP2-eGFP (green). (e) Boxplot comparing the ratio of the intensity of SP-mCherry in the apoplast relative to the inside of the cell. Results represent data from 4 biological repeats, with the following numbers of technical replicates: GFP-HDEL = 23, CXN-GFP = 23, AtLNP1-eGFP = 25 and AtLNP2-eGFP = 17. *** denotes results that are significantly different ($p \leq 0.001$) whilst ns denotes not significantly different results. Scale bars = 5 μm.

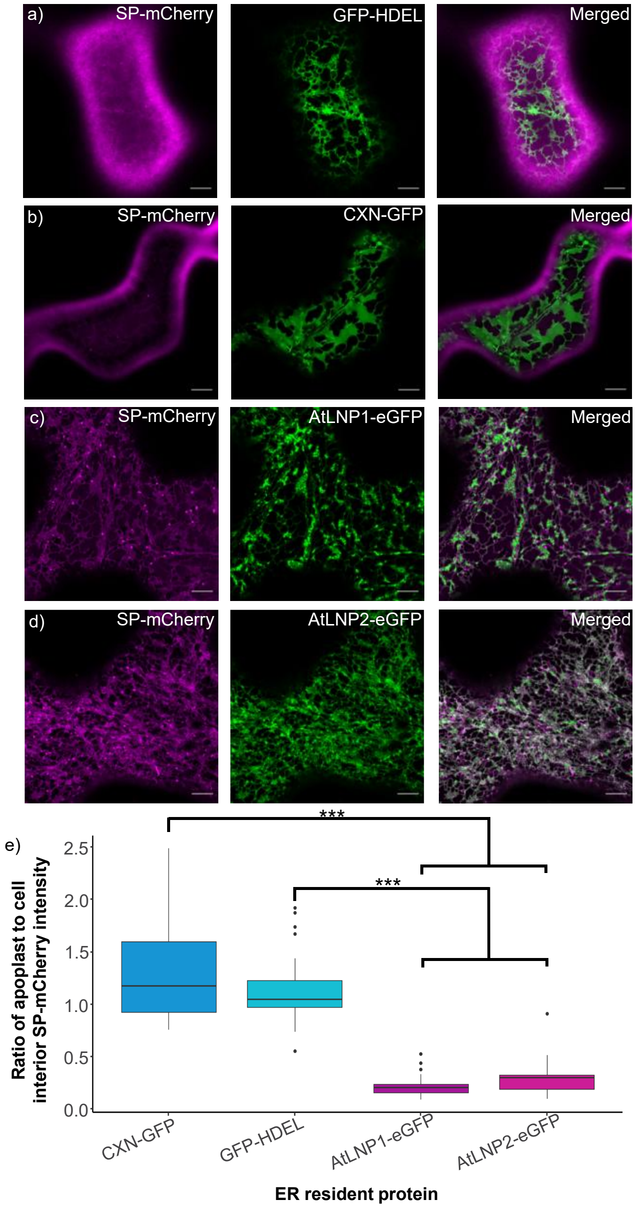

Supplementary Figure S9: Comparison of mean root length of Arabidopsis lines after 10 days of growth.

Boxplot of the root length of two control Arabidopsis lines expressing the ER lumenal marker GFP-HDEL, the ER membrane marker CXN-GFP alongside lines over-expressing AtLNP1-eGFP and AtLNP2-eGFP. Results are shown for GFP-HDEL, n=12; CXN-GFP, n=24; AtLNP1-eGFP, n=29 and AtLNP2-eGFP, n=28 plants. Boxplots show median (centre line), interquartile range (box), and whiskers extending to 1.5× IQR; outliers are plotted as individual points.

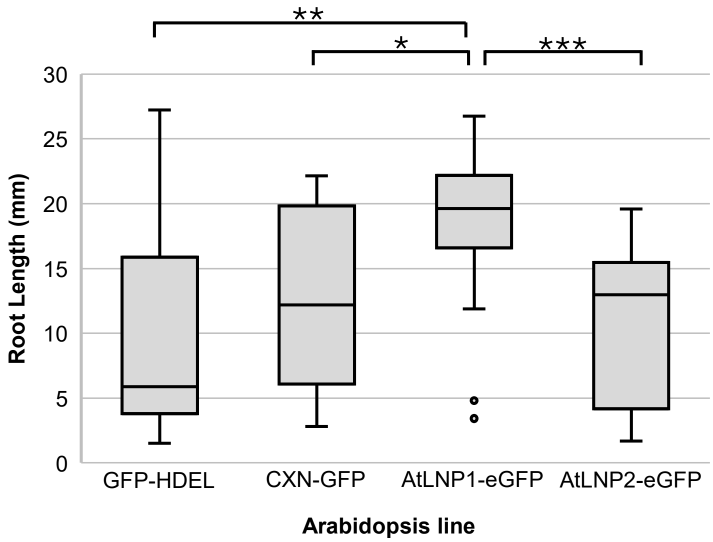

References:

Brandizzi, F., S. Hanton, L. L. DaSilva, P. Boevink, D. Evans, K. Oparka, J. Denecke and C. Hawes (2003). "ER quality control can lead to retrograde transport from the ER lumen to the cytosol and the nucleoplasm in plants." Plant J **34**(3): 269-281.

Groves, N. R., J. F. McKenna, D. E. Evans, K. Graumann and I. Meier (2019). "A nuclear localization signal targets tail-anchored membrane proteins to the inner nuclear envelope in plants." Journal of Cell Science **132**(7): 1-14.

Irons, S. L., D. E. Evans and F. Brandizzi (2003). "The first 238 amino acids of the human lamin B receptor are targeted to the nuclear envelope in plants." Journal of Experimental Botany **54**(384): 93-950.

Kriechbaumer, V., E. Breeze, C. Pain, F. Tolmie, L. Frigerio and C. Hawes (2018). "Arabidopsis Lunapark proteins are involved in ER cisternae formation." New Phytol **219**(3): 990-1004.

Pain, C., V. Kriechbaumer, M. Kittelmann, C. Hawes and M. Fricker (2019). "Quantitative analysis of plant ER architecture and dynamics." Nat Commun **10**(1): 984.

Saint-Jore, C. M., J. Evins, H. Batoko, F. Brandizzi, I. Moore and C. Hawes (2002). "Redistribution of membrane proteins between the Golgi apparatus and endoplasmic reticulum in plants is reversible and not dependent on cytoskeletal networks." The Plant Journal **29**(5): 661-678.

Schwarzländer, M., M. D. Fricker, C. Müller, L. Marty, T. Brach, J. Novak, L. J. Sweetlove, R. Hell and A. J. Meyer (2008). "Confocal imaging of glutathione redox potential in living plant cells." Journal of Microscopy **231**(2).

Wang, P., E. Hummel, A. Osterrieder, A. J. Meyer, L. Frigerio, I. Sparkes and C. Hawes (2011). "KMS1 and KMS2, two plant endoplasmic reticulum proteins involved in the early secretory pathway." The Plant Journal **66**(4).

Zheng, H., L. Kunst, C. Hawes and I. Moore (2004). "A GFP-based assay reveals a role for RHD3 in transport between the endoplasmic reticulum and Golgi apparatus." The Plant Journal **37**(3): 398-414.
